# Genomic Signature of Kin Selection in an Ant with Obligately Sterile Workers

**DOI:** 10.1093/molbev/msx123

**Published:** 2017-04-13

**Authors:** Michael R. Warner, Alexander S. Mikheyev, Timothy A. Linksvayer

**Affiliations:** 1 Department of Biology, University of Pennsylvania, Philadelphia, PA; 2 Ecology and Evolution Unit, Okinawa Institute of Science and Technology, Onna-son, Okinawa, Japan

**Keywords:** kin selection, social evolution, reproductive caste

## Abstract

Kin selection is thought to drive the evolution of cooperation and conflict, but the specific genes and genome-wide patterns shaped by kin selection are unknown. We identified thousands of genes associated with the sterile ant worker caste, the archetype of an altruistic phenotype shaped by kin selection, and then used population and comparative genomic approaches to study patterns of molecular evolution at these genes. Consistent with population genetic theoretical predictions, worker-upregulated genes experienced reduced selection compared with genes upregulated in reproductive castes. Worker-upregulated genes included more taxonomically restricted genes, indicating that the worker caste has recruited more novel genes, yet these genes also experienced reduced selection. Our study identifies a putative genomic signature of kin selection and helps to integrate emerging sociogenomic data with longstanding social evolution theory.

Kin selection theory provides the dominant framework for understanding the evolution of diverse types of social behavior, from cooperation to conflict, across the tree of life (Hamilton 1964; Bourke 2011). While kin selection theory has always had an explicit genetic focus (Hamilton 1964), researchers have made little progress in identifying specific genes that have been shaped by kin selection (Thompson et al. 2013; Ronai et al. 2016), or in identifying genome-wide evolutionary signatures of kin selection (Van Dyken and Wade 2012; Ostrowski et al. 2015). This shortfall is particularly notable in the social insects because the sterile worker caste is the archetypical example of an altruistic phenotype that evolved through kin selection (Hamilton 1964; Queller and Strassmann 1998; Bourke 2011).

The caste system of division of labor between reproductive queens and sterile workers, which first evolved in ants over 100 mya (Ward 2014), is a striking evolutionary innovation that enabled the radiation and ecological dominance of insect societies (Hölldobler and Wilson 1990). While queen and worker castes share the same genome, they express alternate suites of derived traits associated with specialization on either reproduction, or on foraging, nest defense, and brood care (Hölldobler and Wilson 1990). Because queens (and their short-lived male mates) reproduce and hence can directly pass their genes to the next generation, their traits are shaped directly by natural selection. In contrast, obligately sterile workers can only pass on their genes indirectly, by helping their fully fertile relatives to reproduce, so that worker traits are shaped indirectly, by kin selection (Hamilton 1964; Bourke 2011). Population genetic models show that in theory, all-else-equal, genes associated with the expression of worker traits should experience effectively reduced selection (i.e., both reduced positive selection and reduced purifying selection) and correspondingly more nearly neutral molecular evolution when compared with genes associated with the expression of reproductive traits (Linksvayer and Wade 2009, 2016). The relative effective strength of kin selection experienced by worker-associated genes compared with direct selection experienced by reproductive-associated genes should depend on the relatedness between workers and their fully fertile relatives (Linksvayer and Wade 2009; Hall and Goodisman 2012; Linksvayer and Wade 2016).

Using the pharaoh ant, *Monomorium pharaonis*, a derived ant with obligately sterile workers and many queens per colony (i.e., low within-colony relatedness) (Hölldobler and Wilson 1990), in which signatures of kin selection compared with direct selection are expected to be pronounced (Linksvayer and Wade 2009; Linksvayer and Wade 2016), we identified caste-associated genes and studied genomic signatures of short- and long-term molecular evolution of these genes. We used a large set of *M. pharaonis* samples (159 total RNA sequencing libraries; supplementary table S1 and fig. S1, Supplementary Material online) that included a time series of developing worker and reproductive (i.e., queen and male) larvae as well as adult worker and queen head and abdominal tissue (fig. 1*A*) to identify genes that were upregulated in reproductive versus worker castes. The number of differentially expressed genes between worker and reproductive larvae at each stage increased across larval development, corresponding to divergence for overall body size (fig. 1*B*, supplementary fig. S6, Supplementary Material online). Most differentially expressed genes were detected between adult queen and worker abdominal tissue (fig. 1*B*), which is expected given that queens have well-developed ovaries in their abdomens while *M. pharaonis* workers lack reproductive organs.

**Fig. 1:**
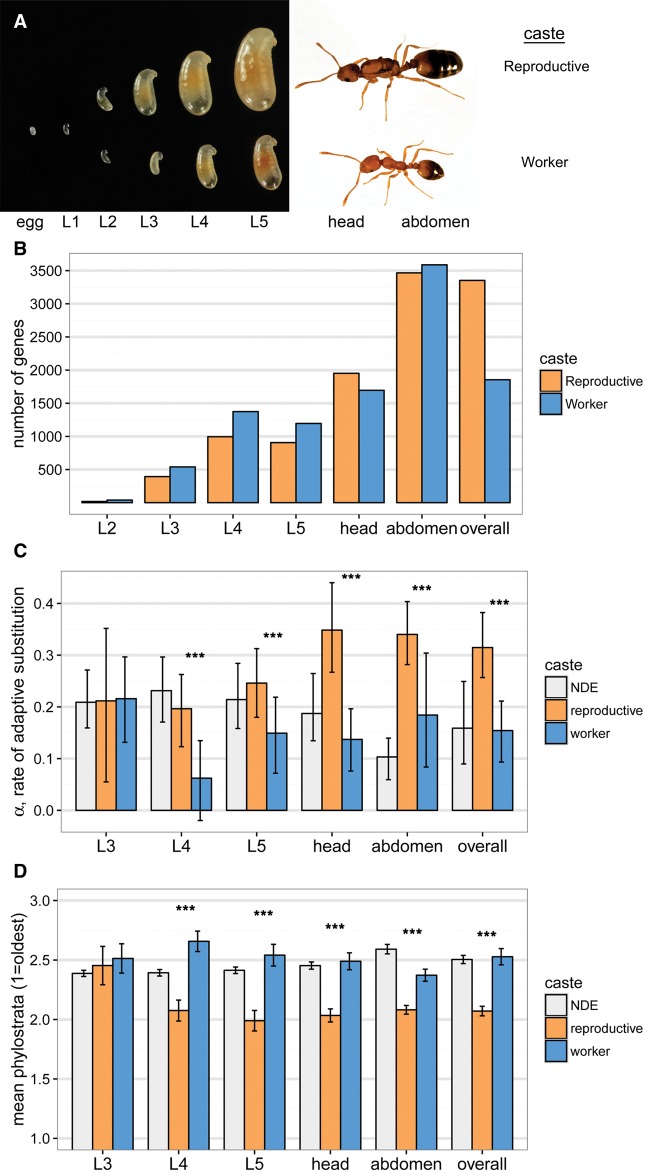
Genomic signature of kin selection. (*A*) In order to identify genes upregulated in reproductives versus worker castes, which should be shaped mainly by direct versus indirect (i.e., kin) selection, respectively, we collected a time series of worker- and reproductive (i.e., queen and male) larvae (L2–L5), as well as adult worker and queen head and abdomen tissue samples. (*B*) Dozens to thousands of genes were differentially expressed and upregulated in either reproductive (orange) or worker (blue) castes for each larval stage and adult tissue sample. “Overall” shows genes that were differentially expressed across all samples (i.e., genes with a main effect of caste on expression). The L2 comparison is excluded from subsequent analyses because only 59 total genes were differentially expressed at this early stage. (*C*) Reproductive-upregulated genes had higher α, the proportion of amino acid substitutions fixed by positive selection, for all comparisons except for L3. NDE, nondifferentially expressed genes. (*D*) Reproductive-upregulated genes were also older on average (i.e., lower mean phylostrata) for all comparisons except L3. The phylostrata were grouped into the six categories as shown in figure 2*A*, but using all original 19 categories produced the same result (supplementary fig. S7, Supplementary Material online). ***P < 0.001.

Next, to compare rates of adaptive molecular evolution at the identified worker- and reproductive-associated genes, we used a population genomic data set based on 22 resequenced *M. pharaonis* worker genomes together with a single *M. chinense* worker genome as an outgroup. We estimated α, the proportion of amino acid substitutions fixed by positive selection (Bierne and Eyre-Walker 2004; Welch 2006; Obbard et al. 2009). This estimated proportion for worker-associated genes (0.15, 95% CI 0.09–0.21) is approximately half that of the estimate for reproductive-associated genes (0.31, 95% CI 0.26–0.38; bootstrap *P* < 0.001; fig. 1*C*), indicating reduced selection experienced by worker-associated genes. Estimates of mean per locus selection and selective constraint parameters (supplementary fig. S5, Supplementary Material online), which are meant to quantify positive and purifying selection, respectively, also supported the conclusion that worker-associated genes have experienced reduced selection (i.e., more nearly neutral molecular evolution) compared with reproductive-associated genes. These results are consistent with theoretical expectations (Linksvayer and Wade 2009, 2016), providing a putative genomic signature of kin selection.

To further elucidate the evolution and genomic basis of ant caste, we used a comparative genomic approach, phylostratigraphy (Domazet-Loso et al. 2007), which estimates the evolutionary age of genes based on whether orthologs can be identified across different strata of the tree of life (i.e., phylostrata). Most of the identified worker- and reproductive-associated genes were ancient (i.e., shared across all cellular organisms, eukaryotes, or bilaterian animals; fig. 2*A*), arising long before the evolution of eusociality, consistent with nondifferentially expressed genes in the *M. pharaonis* genome (fig. 2*A*) as well as better annotated insect genomes (supplementary fig. S7, Supplementary Material online). Thus, the evolutionary origin and elaboration of ant caste seems to largely involve the recruitment of ancient genes, as proposed in a series of hypotheses (West-Eberhard 1996; Amdam et al. 2004; Linksvayer and Wade 2005; Amdam et al. 2006; Toth and Robinson 2007). Reproductive-associated genes, which are mainly composed of genes upregulated in adult queen tissues (supplementary fig. S4, Supplementary Material online), were especially enriched for ancient phylostrata, indicating that the evolution of the queen caste mainly involved the recruitment and long-term conservation of ancient genes involved in cellular functions (supplementary tables S5 and S6, Supplementary Material online). In contrast, worker-associated genes were younger on average than reproductive-associated genes (fig. 1*D*, supplementary fig. S8, Supplementary Material online)(glm, *z* = 10.3, df = 12622, *P* < 0.001), with a relatively larger proportion of genes in younger phylostrata, indicating less long-term conservation of ancient genes and more gene turnover or recruitment of novel genes for the worker caste (fig. 2*A*, supplementary figs. S8 and S9, Supplementary Material online; see also Johnson and Tsutsui 2011; Feldmeyer et al. 2014; Harpur et al. 2014). Interestingly, worker-associated genes in the youngest phylostrata (hymenopteran- and ant-specific genes, which were enriched for chemosensory Gene Ontology categories, supplementary table S6, Supplementary Material online) could putatively underlie ant worker-specific adaptations, however, these genes had α estimates that were not different from zero (bootstrap *P* = 0.86; fig. 2*B*; note that negative α values are caused by sampling error or the presence of mildly deleterious mutations that segregate but do not fix [Obbard et al. 2009]). Thus, the phylostratigraphy results provide further evidence that worker-associated genes experience reduced selection relative to reproductive-associated genes.

**Fig. 2:**
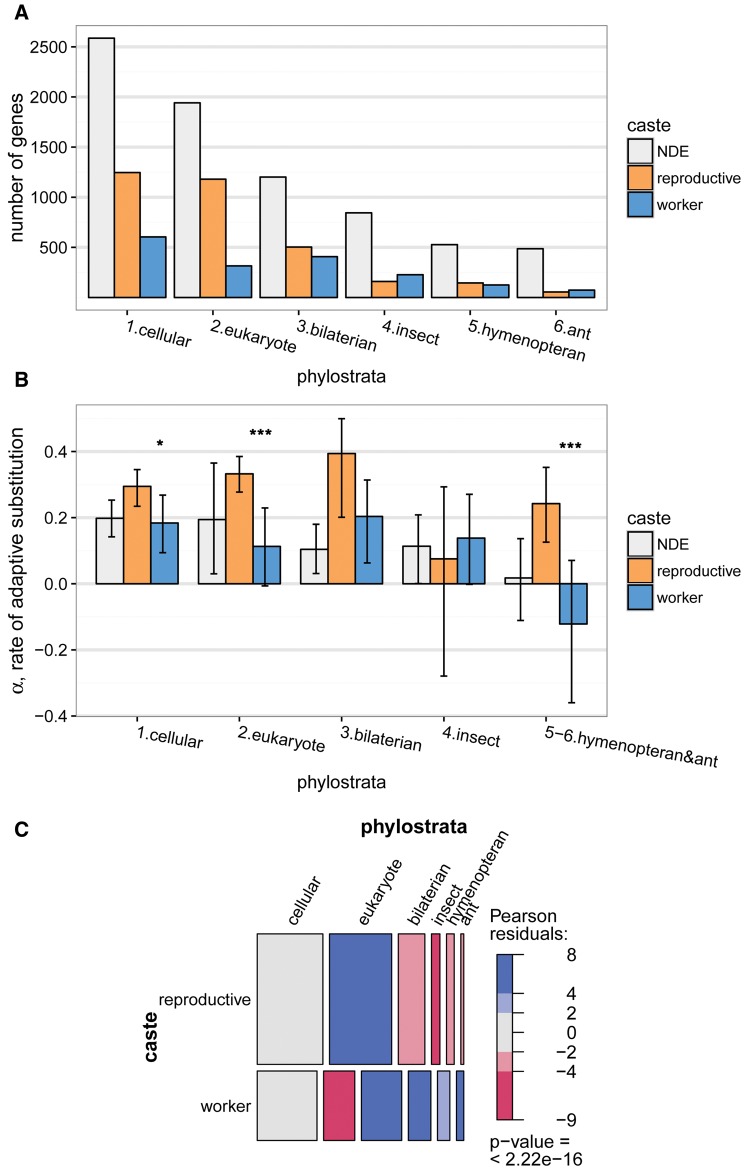
The contribution of ancient and young genes to caste evolution. (*A*) Most caste-associated genes, as well as NDE genes in the *M. pharaonis genome*, are from ancient phylostrata (supplementary fig. S6, Supplementary Material online). (*B*) Genes in the youngest phylostrata tend to show relaxed adaptive evolution (i.e., 95% CI of α overlapping zero), except for reproductive-associated genes in the hymenopteran and ant phylostratum. Genes in the two oldest phylostrata mainly drive the pattern of higher rates of adaptive evolution for reproductive-associated genes relative to worker-associated genes (fig. 1*C*); *P < 0.05, ***P < 0.001. The last two phylostrata (hymenopteran and ant) were combined because there are not enough ant-specific genes for accurate α estimates. (*C*) Mosaic plot showing that relative to reproductive-associated genes, worker-associated genes are enriched for the four youngest phylostrata, while reproductive-upregulated genes are enriched for the eukaryote phylostratum. The area of each cell is proportional to the number of genes in each caste and phylostrata category. Blue shading indicates overrepresentation (light blue *P* < 0.05, dark blue *P* < 0.001), and red-shading indicates underrepresentation (light red *P* < 0.05, dark red, *P* < 0.001), based on cell standardized Pearson residuals.

Recent comparative genomic studies in bees and ants have found signatures of neutral evolution in social insect genomes, thought to be associated with reduced effective population size in species with large societies compared with solitary species (Romiguier et al. 2014; Kapheim et al. 2015). Consistent with these previous findings, our genome-wide estimate of α (0.21, 95% CI 0.17–0.27) is lower than most previous estimates from solitary insects such as *Drosophila* (∼0.5) (Bierne and Eyre-Walker 2004; Welch 2006; Obbard et al. 2009; Galtier 2016; Keightley et al. 2016). Our results suggest that relaxed selection on worker-associated genes also contributes to relatively low genome-wide adaptive substitution rates, so that neutral evolutionary processes are likely to be especially important for worker-associated genes. While it is commonly assumed that intraspecific and interspecific variation for worker morphology and behavior is adaptive (Hölldobler and Wilson 1990; Ferster et al. 2006; Pie and Traniello 2007), our results suggest that relaxed selection and nonadaptive evolutionary forces also play important roles in the evolution of worker traits, in particular for species with low nestmate relatedness (e.g., due to multiple queens or multiple mating; Helanterä et al. 2009).

The population genetic models motivating our study assume that the major difference between genes associated with the expression of worker versus reproductive traits is in having indirect versus direct fitness effects, respectively (Linksvayer and Wade 2009, 2016). Both reproductive and worker castes are essential for colony growth, survival, and reproduction, suggesting strong phenotypic selection on both reproductive and worker traits (Hölldobler and Wilson 1990). However, as far as we know, no empirical estimates exist for the relative magnitude of phenotypic selection on reproductive and worker traits, so that we cannot be sure that that these traits experience similar patterns of phenotypic selection. Thus, besides being a signature of kin selection, the reduced selection we observed at worker-associated genes relative to reproductive-associated genes could also be due to actual reduced phenotypic selection on worker traits compared with reproductive traits (Linksvayer and Wade 2016).

Interestingly, some worker-associated genes in our data set showed evidence of strong positive selection (supplementary fig. S10, Supplementary Material online; supplementary table S8, Supplementary Material online), suggesting that some worker traits may experience strong phenotypic selection (e.g., on the number or survival of new sibling queens) that overcomes the dilution effect of kin selection (Linksvayer and Wade 2009, 2016). In some social insect species, phenotypic selection may even act much more strongly on worker traits than queen traits, as suggested by a recent honey bee population genomic study that found evidence that a set of 90 worker-upregulated genes experienced stronger selection than 79 queen-upregulated genes (Harpur et al. 2014, but see Hunt et al. 2010).

Previous authors have argued that genes experiencing relaxed purifying selection may be more readily recruited into caste (Hunt et al. 2011) and other plastic phenotypes (Leichty et al. 2012), so that elevated rates of molecular evolution at caste-associated genes may be ancestral and not a result of caste-specific evolutionary processes. First, we note that we did not find consistent differences in patterns of molecular evolution between plastic (i.e., the full set of reproductive- plus worker-associated genes) and nondifferentially expressed genes (figs. 1*C* and 2B, supplementary figs. S5 and S6, Supplementary Material online). As stressed above, the clearest and strongest difference we observed was between reproductive-associated and worker-associated genes, while worker-associated genes tended to overlap with nondifferentially expressed genes (fig. 1*C*, supplementary fig. S5, Supplementary Material online). Moreover, because genes in the youngest phylostrata, which are inferred to have evolved in lineages with castes, also show a strong difference between reproductive- and worker-associated genes (fig. 2*B*, supplementary fig. S8, Supplementary Material online), we conclude that social evolutionary processes involved in the origin and elaboration of caste (i.e., kin selection) played an important role in shaping this difference.

Our study identified thousands of genes that have putatively been shaped by kin selection, and hence reveals the promise of identifying genome-wide signatures of social evolution. Our study lends support to the notion that social traits may have distinct genetic and evolutionary features (Mikheyev and Linksvayer 2015), even though the evolution of complex social traits such as caste is mainly based on the recruitment of ancient genes. Our results thus help to tie together previous sociogenomic studies, which have largely been motivated by concepts from Evolutionary Developmental Biology (Toth and Robinson 2007) and have stressed the importance of either highly conserved (Toth and Robinson 2007; Woodard et al. 2011; O’Connell and Hofmann 2012; Berens et al. 2015) or novel genes (Johnson and Tsutsui 2011; Ferreira et al. 2013; Feldmeyer et al. 2014; Sumner 2014; Jasper et al. 2016), with population genetic models based on well-established social evolution theory (Hamilton 1964; Linksvayer and Wade 2009, 2016; Hall and Goodisman 2012).

## Materials and Methods

### Study Design and Sampling Procedure

In order to collect a time series of developing worker and reproductive larvae, and also adult workers and queens, we set up a sacrifice study in which 30 total replicate experimental colonies were randomly assigned to either a queen present or queen absent treatment as well as one of five time points (L1–L5) corresponding to five larval developmental stages, and then sampled at the appropriate time point. Queen removal stimulates the production of new reproductives (i.e., new queens and males) (Edwards 1987; Schmidt et al. 2010) so that following queen removal, a portion of young brood (eggs and first instar larvae) are reared as reproductives, whereas all older brood are reared as workers.

The timing of sampling for colonies in both treatments was based on the current age of the youngest larvae present in the queen removed treatment colonies, which corresponded to brood that were eggs at the time of queen removal. Thus, we sampled the first set of colonies assigned to stage L1 approximately 5 days after creation, at which point nearly all eggs in queen removed colonies had hatched into first instar larvae. Colonies assigned to subsequent stages (L2–L5) were sampled in intervals of 3–4 days, yielding samples of colonies with L2, L3, L4, and L5 larvae. We collected the following samples from each colony: for queen present colonies, we collected worker larvae, adult worker foragers, and adult worker nurses; for queen absent colonies, we collected both worker and reproductive larvae, adult worker foragers, and adult worker nurses observed feeding worker larvae as well as adult worker nurses observed feeding reproductive larvae.

Ten individuals of each sample type were collected and pooled into a single sample. Each individual was immediately flash-frozen in liquid nitrogen after collection. Adult worker heads and gasters (i.e., the last four abdominal segments) were collected separately and removed from the body while frozen. To collect adult queen head and gaster samples, ten mature egg-laying queens ∼4-months-old were collected from three of the genetically homogeneous stock colonies used to create the experimental colonies and processed in the same manner as adult worker samples.

### RNA Sequencing and Mapping

We extracted RNA using RNeasy kits in accordance with manufacturer’s instructions. 25 samples were removed due to contamination or degradation, as detected by an Agilent 2100 Bioanalyzer or poor yield (<50 ng RNA). After excluding these samples, we prepared 161 cDNA sequencing libraries using poly-T capture of messenger RNA and subsequent full-length amplification, as in Aird *et al.* (2013). For quality control and to estimate the dynamic range of the sequencing experiment, we added two ERCC92 (Thermo Fisher Scientific Inc.) spike-in mixes to total RNA, with half the samples randomly receiving one or the other mix. Sequencing of the cDNA libraries was performed on an Illumina HiSeq 2000 in SE50 mode at the Okinawa Institute of Science and Technology Sequencing Center. Reads were mapped to the assembly and NCBI version 2.0 gene models (Mikheyev and Linksvayer 2015) using RSEM (Li and Dewey 2011) to obtain expected counts and fragments per kilobase mapped (FPKM).

### Differential Expression Analysis

We removed genes with FPKM < 1 in at least half the samples of all three tissues (head, gaster, and larvae) from further analysis. We removed two samples from further analysis due to suspected contamination (see supplementary table S1, Supplementary Material online for numbers of samples used for subsequent analysis). We performed differential expression analysis using edgeR (Robinson and Oshlack 2010) with a GLM-like fit to the count data (McCarthy et al. 2012). In order to identify worker-upregulated and reproductive-upregulated genes, we performed differential expression analysis separately by larval stage and adult sample type, across all larval stages, and across all larval stages and adult samples together. We performed subsequent analyses using the sets of genes that had an overall average effect of caste across all larval and adult samples. We assumed that these genes were most tightly associated with worker versus reproductive function, and hence shaped primarily by indirect (i.e., kin) selection versus direct selection.

### Population Genomic Analysis

We constructed genomic sequencing libraries for 22 single-worker specimens of *M. pharaonis* and one outgroup worker sample of *Monomorium chinense*. We chose the ingroup samples to maximize geographic coverage and to provide a representative sample of standing genetic diversity in this species. Sequencing libraries were made using Illumina Nextera kits and sequenced on an Illumina HiSeq 2000 instrument. *M. pharaonis* reads were mapped to the reference using bowtie 2 in very sensitive local mode (Langmead and Salzberg 2012), while the *M. chinense* samples were mapped using NextGenMap (Sedlazeck et al. 2013), which offers more sensitivity for divergent sequences. Subsequently, variants were called separately using GATK, FreeBayes and Samtools (Li et al. 2009; McKenna et al. 2010; Garrison and Marth 2012). These variant call sets were converted to allelic primitives using GATK, and combined into a high credibility set using BAYSIC (Cantarel et al. 2014). We subsequently removed indels, any sites with more than two alleles, with more than 10% missing data, and any with a site quality lower than a phred score of 40 to produce the final variant call set. The effect of each variant (synonymous vs. nonsynonymous) was determined using SnpEff (Cingolani et al. 2012). We then used the resulting table of numbers of synonymous polymorphisms (*P_S_*) and substitutions (*D_S_*) and nonsynonymous polymorphisms (*P_N_*) and substitutions (*D_N_*) for input for McDonald–Kreitman (McDonald and Kreitman 1991) test-based software for estimating population genetic parameters and inferring signatures of selection (Welch 2006; Eilertson et al. 2012).

The McDonald–Kreitman test can be extended to estimate α, the proportion of amino acid substitutions that are fixed by positive selection (Smith and Eyre-Walker 2002; Bierne and Eyre-Walker 2004; Welch 2006), as a powerful way to study genome-wide rates of adaptive molecular evolution. We estimated α for worker-upregulated, reproductive-upregulated, and nondifferentially expressed genes. We used a maximum likelihood estimator developed in the software package MKtest2.0 (Welch 2006; Obbard et al. 2009) (available at http://sitka.gen.cam.ac.uk/research/welch/GroupPage/Software.html, last accessed 1 July, 2016).

### Comparative Genomic Phylostratigraphy Analysis

We constructed phylostratigraphic maps for *M. pharaonis*, as well as two species with higher quality genomes (*Apis mellifera*, and *Drosophila melanogaster*), following previously developed methods (Domazet-Loso et al. 2007; Domazet-Lošo and Tautz 2010; Quint et al. 2012; Drost et al. 2015). Phylostrata were defined for each species according to the NCBI taxonomy database (supplementary table S4 and fig. S7, Supplementary Material online). We constructed a target database by adding recently sequenced hymenopteran genomes to a database that was recently used in a phylostratigraphy study of animal and plant development (Drost et al. 2015). Species-specific amino acid sequences were downloaded from RefSeq (Pruitt et al. 2012), last accessed 11 July, 2016. Each amino acid sequence at least 30 amino acids long for each of the three species were used as a query against the target database using BLASTp (version 2.2.25). Transcripts were assigned to the oldest phylostrata containing at least one BLAST hit with an *E*-value below 10^−5^ for the given transcript. If no BLAST hit with an *E*-value below 10^−5^ was found, the transcript was placed in the youngest, species-specific phylostrata (e.g., *Monomorium pharaonis*). Genes were assigned to phylostrata based on the phylostrata of their longest transcript isoform. To verify that our results did not depend on the *E*-value threshold we used, we also constructed a map for *M. pharaonis* using a very liberal *E*-value threshold of 10^−1^ as well as the default, much more conservative 10^−5^ threshold (Quint et al. 2012).

To compare mean phylostrata between queen- and worker-associated genes, we used generalized linear models with Poisson residuals (or quasi-Poisson for overdispersed models). We used both raw phylostrata (after removing any phylostrata with zero genes; PS in supplementary table S4, Supplementary Material online), as well as phylostrata condensed into 6 main categories because many categories had few genes (supplementary fig. S7, Supplementary Material online): cellular organisms, eukaryotes, bilaterian animals, insects, hymenopterans, and ants (“Condensed PS1” supplementary table S4, Supplementary Material online). To compare the relative contribution of phylostrata to worker- and reproductive-associated genes, we constructed contingency tables, used omnibus Chi-square tests, calculated standardized Pearson’s residuals to explore the contribution of each cell to the omnibus test, and presented the results using mosaic plots with the “vcd” R package (Friendly and Meyer 2015) and the six Condensed PS1 categories. The significance of enrichment for individual cells was assessed with standardized Pearson residuals, where residuals with an absolute value > 2 have an approximate *P*-value < 0.05, and residuals with an absolute value > 4 have an approximate *P*-value < 0.001 (Friendly 1994; Friendly and Meyer 2015).

### Gene Ontology Enrichment Analysis

We calculated GO term enrichment of categories of identified worker-associated and reproductive-associated genes, as well as worker-associated and reproductive-associated genes grouped by phylostrata, using the R package “GOstats”, with a cut-off *P*-value of 0.05 (Falcon and Gentleman 2007).

### Statistical Analyses

All statistical analyses and figures were made with R version 3.1.2.

### Data Deposition

Raw sequencing reads will be deposited in DDBJ bioproject PRJDB3164. Count and FPKM data are available at https://sites.sas.upenn.edu/linksvayer-lab/data, and a .xls file summarizing all analyses for each locus is available as supplementary materials, Supplementary Material online.

## Supplementary Material

Supplementary Data
