# Supplementary material for: Genomic Signature of Kin Selection in an Ant with Obligately Sterile Workers
Source: Mol Biol Evol. 2017 Apr 13;34(7):1780–7. doi: 10.1093/molbev/msx123 (PMC5455959; doi:10.1093/molbev/msx123)
Supplement: Supplementary Data [file msx123_Supp.zip › combined supplement updated 27Mar2017.pdf]

Supplementary Material

Supplemental Methods

Study species

Monomorium pharaonis has the following suite of traits that make it suitable for the current study: unlike most ants and other hymenopteran social insects, which have facultatively sterile workers that can lay male-destined eggs under some conditions, *Monomorium* workers are obligately sterile (Hölldobler and Wilson 1990), so that genes exclusively expressed by *M. pharaonis* workers can only have indirect fitness effects; *M. pharaonis* colonies are readily experimentally induced to shift from producing only new workers, to producing a mixture of new workers and reproductives (i.e. queens and males), by removing current egg-laying queens (Edwards 1987; Schmidt et al. 2010); worker- and reproductive-destined larvae can be morphologically distinguished at an early developmental stage (Fig. 1A) (Berndt and Kremer 1986); controlled crosses can readily be made in the lab and hundreds of colonies kept across generations; and aggression between workers from different colonies is transient so that the genetic makeup of colonies can be experimentally controlled.

Study design and colony setup

For an overview of the study design, see Figure S1. The study was run in three total blocks (yielding three biological replicates), each separated by three weeks, starting in April 2014. For each block, we did the following:

1. We created a genetically homogeneous source by mixing at least 10 large stock colonies, which themselves had been repeatedly mixed across generations (note that unlike most ants, *M. pharaonis* colonies display at most transient aggression following colony mixing).

2. From this source, we allocated 0.5 mL of mixed brood and workers to each replicate experimental colony, resulting in colonies with ~300-400 workers and ~300-400 brood of various stages (i.e. eggs, larvae, pupae).

3. We randomly assigned half of the experimental colonies to a queen present treatment, where queen number was standardized to 10 queens, and the other half to a queen absent

treatment, where all queens were removed. Queen removal stimulates the production of new reproductives (i.e. new queens and males) (Edwards 1987; Schmidt et al. 2010) so that following queen removal, a portion of young brood (eggs and 1st instar larvae) are reared as reproductives, whereas all older brood are reared as workers.

4. We also randomly assigned each experimental colony to one of five time points (L1-L5), corresponding to five larval developmental stages (see below).

All colonies were maintained at 27 ± 1 ºC and 50% humidity, and fed twice weekly with dried mealworms (*Tenebrio molitor*) and an agar-based synthetic diet (Dussutour and Simpson 2008). Experimental colonies were maintained in glass nests made of two pieces of 4 cm x 6 cm glass separated by 1.5 mm strips of plastic. All surveys and sampling were performed using dissecting microscopes.

Sampling procedure

Note that adult *M. pharaonis* workers performing foraging or nursing tasks have been shown to have different whole-body gene expression profiles (Mikheyev and Linksvayer 2015), and we collected forager and nurse samples across social contexts (queen presence and larval stage) in order to be able to confidently identify genes that are consistently upregulated in worker tissues.

On the day designated for sample collection, colonies were placed in petri dishes, surveyed, and then lightly anesthetized using carbon dioxide to prepare the colony for sample collection. While anesthetized, the top glass pane of the nest was removed to enable collection of nursing workers, the petri dish was covered with a lid to minimize disturbance from air flow, and the petri dish was placed on a heating pad, kept at 27 ºC, to maintain a constant temperature throughout sample collection. Colonies were left undisturbed for 30 minutes to recover from anesthesia prior to sample collection. Foragers were collected when observed collecting food outside the nest, and nurses were collected when observed nursing the appropriate larval stage. For example, for the L2 sample, worker nurses were collected when witnessed feeding a 2nd instar worker larva, and reproductive nurses when witnessed feeding a 2nd instar reproductive larva. After all foragers and nurses were collected, colonies were lightly anesthetized again and larvae of the appropriate stage were collected.

Larval instars were determined by overall size, shape, and especially hair presence and

morphology (Berndt and Kremer 1986). *M. pharaonis* larvae have three distinct larval instars (Berndt and Kremer 1986), but because the vast majority of growth occurs in the third larval instar, we divided the third instar into three separate stages (L3-L5) based on size (Fig. 1A). Third instar worker larvae were defined as the L3 stage until they reached 0.75x the length of a worker pupa, L4 stage up to 1x the length of worker pupae, and L5 thereafter. Reproductive larvae are hairless (Berndt and Kremer 1986) and can be differentiated from worker larvae starting at the 2nd larval instar. Reproductive larval stages were defined as follows: 2nd instar (i.e. L2) until 0.5x the length of a worker pupa, L3 from 0.5-1x the length of a worker pupae, L4 from 1-1.5x the length of a worker pupa, and L5 thereafter.

Note that it is not possible to morphologically distinguish between male and queen larvae, so that the reproductive larvae we collected included both males and queens. *M. pharaonis* colonies produce female-biased reproductive sex ratios (e.g., 0.739 female [queen/(queen+male)], interquartile range = 0.028, based on 39 colonies, (Schmidt et al. 2010)), and furthermore, newly eclosed adult queens are on average 1.42 times as large as males (1.359 mg wet mass versus 0.955 mg, based on samples of 247 newly eclosed queens and 235 males, (Schmidt et al. 2010)). Thus, we estimate that approximately 80% of the sampled larval tissue came from queens, so that the transcriptomic profiles we observed for our reproductive larvae samples mostly reflected queen larvae.

We separately collected adult head and abdominal tissues to increase the likelihood of detecting differentially expressed genes associated with adult behavior (e.g., genes upregulated in worker brains) and function (e.g., genes upregulated in queen reproductive tissues).

Differential expression analysis

For stage-specific analyses, caste was the only factor included. For analyses including all larval samples or all adult and larval samples, we used a model with caste and stage as fixed factors. For comparisons only considering larval stages, we included batch as an additional factor, but we could not include batch in comparisons including adult samples because the queen samples were not collected within the same blocked design.

Population genomic analyses

The McDonald-Kreitman test (McDonald and Kreitman 1991) uses both polymorphism and substitution data (e.g., an excess of nonsynonymous substitutions relative to polymorphisms; $D_N/D_S > P_N/P_S$) to infer the fixation of advantageous mutations by positive selection (Bierne and Eyre-Walker 2004). This approach is more powerful at identifying signatures of positive selection than using only substitution data for divergent lineages (i.e. with d_N/d_S, which is D_N/D_S weighted by the total numbers of nonsynoymous and synonymous sites), because elevated d_N/d_S estimates can arise either from positive selection or relaxed purifying selection.

MKtest2.0 was especially suitable for our needs because it can simultaneously estimate α for different gene categories (Obbard et al. 2009), instead of only providing a single genome-wide estimate. Besides estimating α, MKtest2.0 can estimate other population genetic parameters, including selective constraint, described by 1-*f*, the proportion of non-synonymous mutations that experience strong purifying selection, as well as neutral diversity, and neutral divergence. Because we were interested in comparing patterns of selection experienced by worker- and reproductive-upregulated genes, and to avoid overparameterized models, we focused on α and *f* and kept other parameters at default values (i.e. a single global value). We compared model estimates for α and *f* and model fit statistics using a single genome-wide estimate (i.e. the default), separate estimates for each of three categories (i.e. reproductive-associated, worker-associated, and NDE), and for *f*, we also considered separate estimates for each locus. Because models with gene-specific *f* estimates were best (Table S6), we focus on the estimates from these models in the main text, although we observed similar patterns for other parameters as well. We estimated 95% confidence intervals for α with the bootstrapping feature in MKtest2.0 (i.e. as 95% bootstrap intervals around the mean, based on 1,000 bootstrap replicates across genes). We also used bootstrapping to determine p-values for the hypothesis that reproductive-associated genes have α greater than worker-associated genes. Similarly, we used bootstrapping to determine p-values for the hypothesis that caste-associated genes grouped by phylostrata had α greater than zero. We used beta regression (R package "betareg"; (Cribari-Neto and Zeileis 2010)), to compare mean *f* estimates between worker-associated and queen-associate genes.

In addition to estimating α across groups of genes in order to compare rates of adaptive

molecular evolution at these genes, it is possible to estimate selection coefficients separately for each locus and then compare mean estimated selection coefficients. The software SnIPRE (Eilertson et al. 2012), Selection Inference using Poisson Random Effects, is a Bayesian implementation of the McDonald-Kreitman test and seeks to estimate several population genetic parameters similar to those estimated by MKtest2.0, including the selection coefficient acting on every gene weighted by effective population size ($\gamma = 2N_e s$). We used SnIPRE to estimate γ for each gene and compared mean γ for the categories of caste-associated genes we identified. Specifically, we used a generalized linear model (glm) to compare mean BSnIPRE.est, a normalized estimate of γ produced by SnIPRE, for worker-associated, reproductive-associated, and NDE genes.

We also used SnIPRE to categorize genes as experiencing positive, neutral, or negative selection. We also classified genes as experiencing selection using the standard McDonald-Kreitman test (McDonald and Kreitman 1991), by plotting log(p-value) from this test versus the -log(*NI*) (Li et al. 2008), where NI is the neutrality index. We used an unbiased estimator for NI (Stoletzki and Eyre-Walker 2010). Genes above a threshold p-value from the McDonald-Kreitman test with a positive -log(*NI*) were categorized as having experienced positive selection, and those with a negative -log(*NI*) were categorized as having experienced negative selection (Li et al. 2008). We used both a liberal cutoff of 0.05 for the nominal p-value from the McDonald-Kreitman test, and also a much more conservative Bonferroni-corrected p-value cutoff (Li et al. 2008) based on the 5,674 genes included in the analysis (genes with zeros for any of the four counts D_N,D_S, P_N,P_S were excluded because such zeros lead to undefined *NI*).

<u>Comparative genomic phylostratigraphy analysis</u>

Phylostratigraphy attempts to estimate the evolutionary age of protein-coding genes in a focal species by identifying the distribution of their homologs across the tree of life (Domazet-Loso et al. 2007; Domazet-Lošo and Tautz 2010; Quint et al. 2012; Drost et al. 2015). The approach sorts genes into hierarchical phylostrata (PS), based on the oldest BLAST hit of their amino acid sequence. For example, a gene from an ant species with identified orthologs across all eukaryotes is assumed to be much older than a gene with only identifiable orthologs in other closely related ant species. We were interested in estimating the relative ages of the the sets of worker-

upregulated, reproductive-upregulated, and non-differentially expressed genes that we identified, and were less interested in the precise age estimates. Indeed, even though phylostratigraphy has been widely used (Domazet-Loso et al. 2007; Domazet-Lošo and Tautz 2010; Quint et al. 2012; Drost et al. 2015), the precise age estimates can be influenced by several factors, including parameters used to define homology in BLAST (e.g., threshold gene length, threshold E-value, database size, etc.) (Moyers and Zhang 2016; Moyers and Zhang 2015). Furthermore, because homologous sequences in BLAST are generally required to span relatively small lengths (i.e. 30 amino acids) (Quint et al. 2012; Drost et al. 2015), small portions of a gene can impact the phylostrata assigned.

Subsampling analysis

Because there was large variation in the number of replicates among sample type (Fig. S1, Table S1), we used subsampling to study whether our results depended on which exact samples were analyzed. Specifically, we used a very conservative approach in which we randomly subsampled all sample types 1,000 times so that each sample type had the same sample number, corresponding to the minimum number of samples for any sample type (i.e. n=3 or n=2 for abdominal and head samples, respectively, see Table S1). This conservative subsampling analysis produced a similar overall pattern for alpha as when all samples are analyzed, with queen-upregulated genes having higher estimated alpha compared to worker-upregulated genes, but this pattern was only significant in the subsampled data for head samples (for adult head samples: queen alpha = 0.284 [95% CI 0.155-0.412] versus worker alpha= 0.0941 [-0.00503-0.214], p = 0.0325; for adult abdominal samples, queen alpha = 0.319 [0.126-0.353] versus worker alpha = 0.198 [0.131-0.349], p = 0.350). Thus, our main results are robust to variation in sample size between sample types as well as which exact samples are included in the analysis.

Statistical analyses and figures

All statistical analyses and figures were made with R version 3.1.2, using packages “ggplot2”, ”gplots”, ”scales”, “stats”, “plyr”, “ggdendro”, “gridExtra”, “tidyr”, ”plyr”, ”vcd”, ”vcdExtra”, “Vennerable”, ”data.table”, “edgeR”, “myTAI”, “betareg”, and “GOstats”. Complete R scripts

used in the analyses will be included in the final publication. For Figure 1A, we collected representative worker and reproductive larvae from each stage (Berndt and Kremer 1986) and arranged them in a series in order to produce a figure representing the developmental time series we used.

References

Berndt K, Kremer G. 1986. The morphology of larvae of the pharaoh's ant *Monomorium pharaonis* (L.) (Hymenoptera. Formicidae). Zool. Anz. 216:305–320.

Bierne N, Eyre-Walker A. 2004. The genomic rate of adaptive amino acid substitution in *Drosophila*. Mol. Biol. Evol. 21:1350–1360.

Cribari-Neto F, Zeileis A. 2010. Beta regression in R. J. Stat. Softw. 34:1–24.

Domazet-Loso T, Brajković J, Tautz D. 2007. A phylostratigraphy approach to uncover the genomic history of major adaptations in metazoan lineages. Trends Genet. 23:533–539.

Domazet-Lošo T, Tautz D. 2010. A phylogenetically based transcriptome age index mirrors ontogenetic divergence patterns. Nature 468:815–818.

Drost H-G, Gabel A, Grosse I, Quint M. 2015. Evidence for active maintenance of phylotranscriptomic hourglass patterns in animal and plant embryogenesis. Mol. Biol. Evol. 32:1221–1231.

Dussutour A, Simpson SJ. 2008. Description of a simple synthetic diet for studying nutritional responses in ants. Insectes Soc. 55:329–333.

Edwards JP. 1987. Caste regulation in the pharaoh's ant *Monomorium pharaonis*: the influence of queens on the production of new sexual forms. Physiol. Entomol. 12:31–39.

Eilertson KE, Booth JG, Bustamante CD. 2012. SnIPRE: selection inference using a Poisson random effects model. PLoS Comput. Biol. 8:e1002806.

Hölldobler B, Wilson EO. 1990. The Ants. Cambridge, MA: Harvard University Press

Li YF, Costello JC, Holloway AK, Hahn MW. 2008. "Reverse ecology" and the power of population genomics. Evolution 62:2984–2994.

McDonald JH, Kreitman M. 1991. Adaptive protein evolution at the Adh locus in *Drosophila*. Nature 351:652–654.

Mikheyev AS, Linksvayer TA. 2015. Genes associated with ant social behavior show distinct transcriptional and evolutionary patterns. Elife 4:e04775.

Moyers BA, Zhang J. 2015. Phylostratigraphic bias creates spurious patterns of genome evolution. Mol. Biol. Evol. 32:258–267.

Moyers BA, Zhang J. 2016. Evaluating phylostratigraphic evidence for widespread de novo gene birth in genome evolution. Mol. Biol. Evol. 33:1245–1256.

Obbard DJ, Welch JJ, Kim K-W, Jiggins FM. 2009. Quantifying adaptive evolution in the *Drosophila* immune system. PLoS Genet. 5:e1000698.

Quint M, Drost H-G, Gabel A, Ullrich KK, Bönn M, Grosse I. 2012. A transcriptomic hourglass in plant embryogenesis. Nature 490:98–101.

Schmidt AM, Linksvayer TA, Boomsma JJ, Pedersen JS. 2010. Queen–worker caste ratio depends on colony size in the pharaoh ant (*Monomorium pharaonis*. Insectes Soc. 58:139–144.

Stoletzki N, Eyre-Walker A. 2010. Estimation of the neutrality index. Mol. Biol. Evol. 28:63–70.

Welch JJ. 2006. Estimating the genomewide rate of adaptive protein evolution in *Drosophila*. Genetics 173:821–837.

Figures and Tables

Fig. S1. A. Diagram of the sampling design. 30 replicate colonies were created from a common source and used to create three biological replicates each for the following 10 conditions: one of 5 time points (L1-L5) and two queen presence conditions (Q^-, queen absent; Q^+, queen present). Colonies assigned to the first time point (L1) were sampled when the eggs that were present at the start of the study hatched into first instar larvae. Colonies assigned to subsequent time points (L2-L5) were subsequently sampled when these larvae reached the appropriate developmental stage (see main text for definitions of the stages). For each colony, at the appropriate time (based on whether the colony was assigned to L2-L5), we collected appropriate-stage larvae as well as adult nurse workers observed feeding appropriate-stage larvae and adult nurse foragers. Q+ colonies rear only new workers, while Q- colonies rear both workers and reproductives. Therefore, in Q- colonies, we collected worker larvae (labeled "Larvae (W)") and reproductive larvae ("Larvae (R)"), as well as nurses observed feeding worker larvae ("Nurses(W)") and nurses observed feeding reproductive larvae ("Nurses(R)"), and foragers. In Q+ colonies, we collected worker larvae, nurses observed feeding worker larvae, and foragers. Note that at the L1 stage, it is not possible to morphologically distinguish between

reproductive and worker larvae, so larvae and nurses observed feeding larvae at this stage are marked “W/R”. Adult queens were sampled separately from a common colony source. For larval samples, we used whole bodies, while for all adult samples (nurses, foragers, queens), we separated head and abdominal (i.e. gaster) tissue. We pooled 10 individuals for each sample/tissue type. **B. Summary of total numbers of pooled samples collected for each sample/tissue type.** Our study focused on identifying and studying genes that were differentially expressed between reproductive larvae and adult queens, in comparison with worker larvae and adult workers (see Fig. 1). Thus, during analyses of our adult worker samples, we did not separately consider nurses that were observed feeding worker versus reproductive larvae, or foragers, but only considered adult workers altogether. As a result, our sampling scheme resulted in many more total nurse and forager samples (42 and 30 respectively), compared to worker and reproductive larval samples (24 and 12 respectively), and in particular compared to adult queen samples (3). See also Table S1 detailing the total number of samples per sample/tissue type and condition that were successfully analyzed.

Fig. S2. Log2 fold change (Reproductive/Worker) as a function of the mean of worker and reproductive expression (FPKM) across all larval stages and adult samples. Genes with annotation information and FPKM > 500 and a LogFC of a greater magnitude than 2.5 are labeled. Genes that are colored are differentially expressed, with a main effect of caste across samples: orange = reproductive-upregulated; blue = worker-upregulated; grey = non-differentially expressed (NDE). For plotting purposes, genes with a log2 fold greater than (less than) 5 (-5) assigned a value of 5 (-5). Genes with greater mean expression than 1000 FPKM assigned a value of 1000 FPKM.

Fig S3. Log2 fold change (Reproductive/Worker) as a function of the mean of worker and reproductive expression (FPKM) for specific larval stages or adult samples. Results are from (**A**) across larval stages L2-L5, (**B**) adult head, (**C**) adult gaster, (**D**) L2, (**E**) L3, (**F**) L4, and (**G**) L5. Genes that are colored are differentially expressed, with a main effect of caste across samples: orange = reproductive-upregulated; blue = worker-upregulated; grey = NDE. For plotting purposes, genes with a log2 fold greater than (less than) 5 (-5) were assigned a value of 5 (-5). Genes with greater mean expression than 1000 FPKM were assigned a value of 1000 FPKM.

Fig. S4. Weighted three-set Venn diagrams showing the contribution of: **A.** queen gaster-upregulated genes, queen head-upregulated genes, and the union of all reproductive larvae-upregulated genes (for L2-L5) to the set of reproductive-upregulated genes with a main effect across all samples; **B.** Worker gaster-upregulated genes, worker head-upregulated genes, and the union of all worker larvae-upregulated genes (for L2-L5) to the set of worker-upregulated genes with a main effect across all samples. Note that the set of reproductive-upregulated genes is dominated by genes upregulated in adult queen tissues, with 80% of reproductive-upregulated genes upregulated in queen abdominal (i.e. gaster) tissue, and 46% in queen head tissue. 41% (i.e. 1341/3252) are only upregulated in queen gasters, and not in any other tissue. In contrast, the set of worker-upregulated genes is more evenly composed of genes upregulated in worker gaster, head, and larval samples.

Fig. S5. Estimated per-locus selective constraint and selection parameters across samples. (**A**) Reproductive-upregulated genes with a main effect across all samples ("overall") and reproductive-upregulated genes from queen abdomens had higher mean selective constraint (=lower *f*) than worker-upregulated genes. Locus-specific *f* estimates were made with MKtest2.0. (**B**). Except for the L3 comparison, reproductive-upregulated genes in all comparisons have a higher mean selection parameter, BSnIPRE.est, estimated by SnIPRE (glm using the normalized estimate BSnIPRE.est, which is a normalized estimate of gamma = $2N_e*s$; Eilertson et al. 2012). For L3, worker-associated genes have a higher estimate than reproductive associated genes. * $p < 0.05$, ** $p < 0.01$, *** $p < 0.001$.

Fig. S6. (**A**) Number of differentially expressed genes for only larval samples (L3-L5) as well as "overall larvae", genes with a main effect of caste across larval samples. (**B**) Reproductive-associated genes have higher α, the proportion of amino acid substitutions fixed by positive selection, for larval genes, except at the L3 stage. * $p < 0.05$, ** $p < 0.01$.

Fig. S7. Number of genes in each phylostrata, as defined by the NCBI taxonomy database for: (**A**) *M. pharaonis* (E-value = 1×10^{-5}), (**B**) *M. pharaonis* (E-value = 1×10^{-1}), (**C**) *D. melanogaster* (E-value = 1×10^{-5}), (**D**) *A. mellifera* (E-value = 1×10^{-5}). The overall distribution

is similar for all three species, with the majority of genes being ancient, and the pattern observed for *M. pharaonis* is consistent even when a very liberal threshold (E-value = 1×10^{-1}) is used. BLASTp hits are made against a database containing nearly all species with curated genome annotations, with a minimum match length of 30 amino acids and a maximum E-value as listed above.

Fig. S8. Using the original 19 phylostrata, reproductive-upregulated genes were older on average (i.e. lower mean phylostrata) for all comparisons except L3, the same result as when using grouped phylostrata (Fig. 1D). *** $p<0.001$.

Fig. S9. Mosaic plot showing the relative contribution of phylostrata to sets of reproductive-associated, worker-associated, and NDE genes. As when only considering worker- and reproductive-associated genes (Fig. 2C), reproductive-associated genes are enriched for the eukaryote phylostratum, but also for the cellular organism phylostratum. Similarly, worker-associated genes are enriched for bilaterian animal and insect phylostrata, but relative to NDE genes are no longer significantly enriched for the youngest two phylostrata (hymenopteran and ant) (Fig. 2C). The area of each cell is proportional to the number of genes in each caste and phylostrata category. Blue shading indicates overrepresentation (light blue $p < 0.05$, dark blue $p < 0.001$), and red-shading indicates underrepresentation (light red $p < 0.05$, dark red, $p < 0.001$), based on cell standardized pearson residuals.

Fig. S10. P-value as calculated from the McDonald-Kreitman test plotted against the neutrality index, which has been -log transformed, such that positive values indicate positive selection and negative indicate purifying/balancing selection. The solid black line indicates the nominal p-value (0.05) while the dashed line indicates the p-value after Bonferroni correction (N = 5674). Genes are colored by differential expression: grey = non-differentially expressed; orange = reproductive; blue = worker. For plotting purposes, genes with p-values less than 1×10^{-10} were assigned a p-value of 1×10^{-10}, and those with a -log transformed neutrality index of greater (less) than 3 (-3) were assigned a value of 3 (-3).

Fig. S11. Overlap of A) positively and B) negatively selected genes as defined by SNiPRE and

the Neutrality Index. Genes with negative values of -log10(Neutrality Index) and p-values less than 0.05 (for nominal) are defined by the “NI” method as under purifying selection, while such genes with positive -log10(Neutrality Index) values are assigned to the positive selection category. “NI, B-F correction” uses the same method but the p-value cutoff from the MKtest is adjusted for multiple comparisons using the Bonferroni procedure.

Table S1. The number of biological replicates (out of three initial) for each sample type that were successfully analyzed (note that discrepancies between the study design shown in Figure S1 resulted from sample loss during processing). Please see Sampling Procedure and Fig. S1 for further details. Replicate colonies were assigned to a queen present or queen absent treatment (“Queen Presence” column) as well as to one of five time points (“L1” through “L5” columns). At each time point, larvae, nurses, and foragers were collected. Larvae from the L2-L5 time points were separated based on morphology into worker larvae and reproductive larvae, and we separately collected nurses that were observed feeding worker larvae (= “Worker Nurse”) or reproductive larvae (= “Reproductive Nurse”). Whole larvae were processed, while the heads and gasters (=abdominal tissue) of nurses, foragers, and queens were processed separately. Reproductive larvae are not distinguishable at the L1 stage, hence the “N/A” for reproductive larvae and reproductive nurse samples at the L1 time point. Adult queen samples are in the “Other” column because they were collected separately, after the L1-L5 time points were collected, hence the “N/A” for L1-L5 for adult queen samples.

Table S2. Top 20 worker-upregulated genes, sorted by FDR. Differential expression calculated using glm-like model including caste and developmental stage as fixed effects. Negative values of Log2 fold change indicate higher expression in worker samples.

Table S3. Top 20 reproductive-upregulated genes, sorted by FDR. Positive values of Log2 fold change indicate higher expression in reproductive samples.

Table S4. Summary of the raw phylostrata identified for genes in the *M. pharoanis* genome (Fig.

S6), and 6 categories that phylostrata were grouped into “Condensed PS1”. For some analyses that required ~100 genes in each caste-associated category, we also created a third grouping, “Condensed PS2” that combined the hymenopteran and ant categories.

Table S5. Top 3 GO terms for workers and reproductives for each differential expression test, as calculated using the R package GOstats, sorted by p-value. L2 not included due to paucity of differentially expressed genes.

Table S6. Top 3 GO terms for each phylostrata category for each differential expression test, as calculated using the R package GOstats, sorted by p-value. L2 is not included due to paucity of differentially expressed genes. Missing phylostrata categories returned no significant GO terms.

Table S7. Model selection parameters from MKtest2.0 (Welch 2006; Obbard et al. 2009) for estimating α, the proportion of amino acid substitution driven by positive selection. The first three columns show the number of parameters for α and f, as well as the total number of model parameters, K. We mainly considered models with per-class estimates (i.e. three separate estimates for worker-associated, reproductive-associated, and NDE genes) for both α and f, or with per-locus estimates for f. Of these two main models in bold that we considered, the model including per-locus estimates of f fit the data much better. We focus on results from this model, although the per-class α and f model produced very similar results, showing the same pattern and overlapping α estimates. We also show results from models where α and/or f is fixed or had a single, genome-wide estimate. LnL maximized log likelihood; AIC, Akaike information criterion; AICc, second-order AIC; BIC, Bayesian information criterion (Welch 2006; Obbard et al. 2009).

Table S8. Top 20 positively selected genes (sorted by p-value of McDonald-Kreitman test) for reproductive- and worker-associated genes. SnIPRE.class is the selection categories as calculated by SnIPRE. “NI.class” refers to selection categories, as calculated using a combination of the neutrality index and the P-value from the McDonald-Kreitman test. Genes with negative values of -log10(Neutrality Index) and p-values less than 0.05 are defined as under purifying selection, while such genes with positive -log10(Neutrality Index) values are assigned to the positive

selection category. “NI.class B-F correction” uses the same method but the p-value cutoff from the McDonald-Kreitman test is adjusted for multiple comparisons using the Bonferroni procedure.

External Database S1. Complete list of genes summarizing the per-locus results of differential expression analyses, population genomic analyses, and phylostratigraphy analyses. Columns show: annotation from SwissProt and UniProt; results from differential expression analysis by larval stage (L2-L5), adult head and gaster (abdominal) tissue, across all larval samples, and across all samples, with levels NDE = non differentially expressed genes, Reproductive = reproductive-upregulated, and Worker = worker-upregulated; counts of nonsynonymous and synonymous polymorphisms within *M. pharaonis* and fixed differences between *M. pharaonis* and *M. chinense*, and total numbers of nonsynonymous and synonymous sites; results from SnIPRE analysis including BSnIPRE.class, whether genes are categorized by SnIPRE as experiencing positive selection (“pos”), negative selection (“neg”), or neither (“neut”), BSnIPRE.gamma, a population-size calibrated selection coefficient estimate, and BSnIPRE.est, normalized BSnIPRE.gamma; “NI.class” refers to selection categories, as calculated using a combination of the neutrality index and the p-value from the McDonald-Kreitman test: Genes with negative values of -log10(Neutrality Index) and p-values less than 0.05 are defined as under purifying selection, while such genes with positive -log10(Neutrality Index) values are assigned to the positive selection category. “NI.class B-F correction” uses the same method but the p-value cutoff from the McDonald-Kreitman is adjusted for multiple comparisons using the Bonferroni procedure; Finally, the assigned raw (“Raw PS”) and condensed phylostrata (“PS1” and “PS2”; Table S4) from the phylostratigraphy analyses are shown.

External Database S2. Complete GO enrichment analysis results for workers and reproductives for each differential expression test, as calculated using the R package GOstats, sorted by p-value. L2 not included due to paucity of differentially expressed genes.

External Database S3. Complete GO enrichment analysis results for each phylostrata category for each differential expression test, as calculated using the R package GOstats, sorted by p-value. L2 not included due to paucity of differentially expressed genes. Missing phylostrata

categories returned no significant GO terms.

External Database S4. Raw counts per locus from RNA sequencing showing level of expression across all samples included in the study (Table S1).

External Database S5. Raw FPKM per locus from RNA sequencing showing level of expression across all samples included in the study (Table S1).

Figure S1

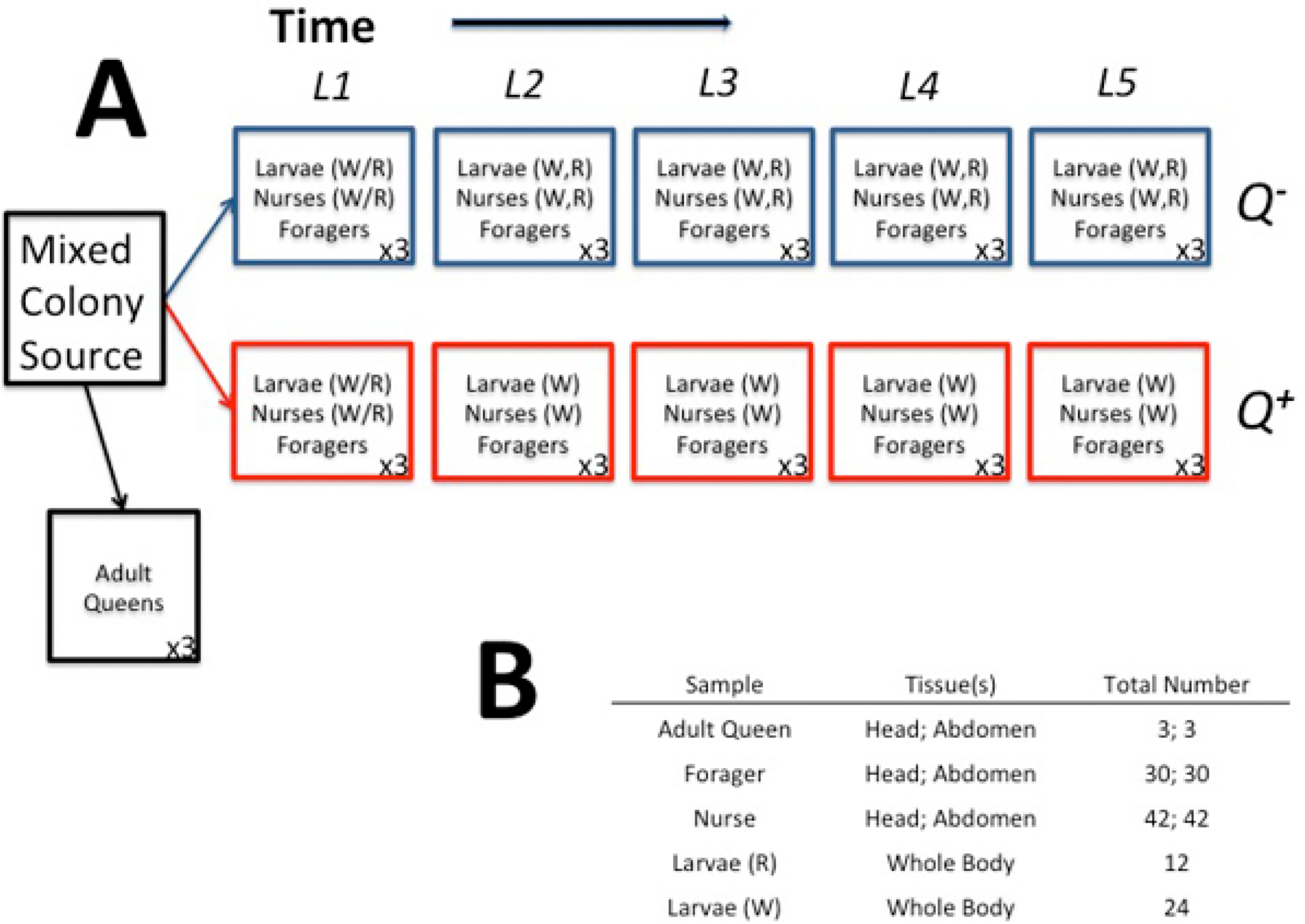

B

Sample	Tissue(s)	Total Number
Adult Queen	Head; Abdomen	3; 3
Forager	Head; Abdomen	30; 30
Nurse	Head; Abdomen	42; 42
Larvae (R)	Whole Body	12
Larvae (W)	Whole Body	24

Figure S2

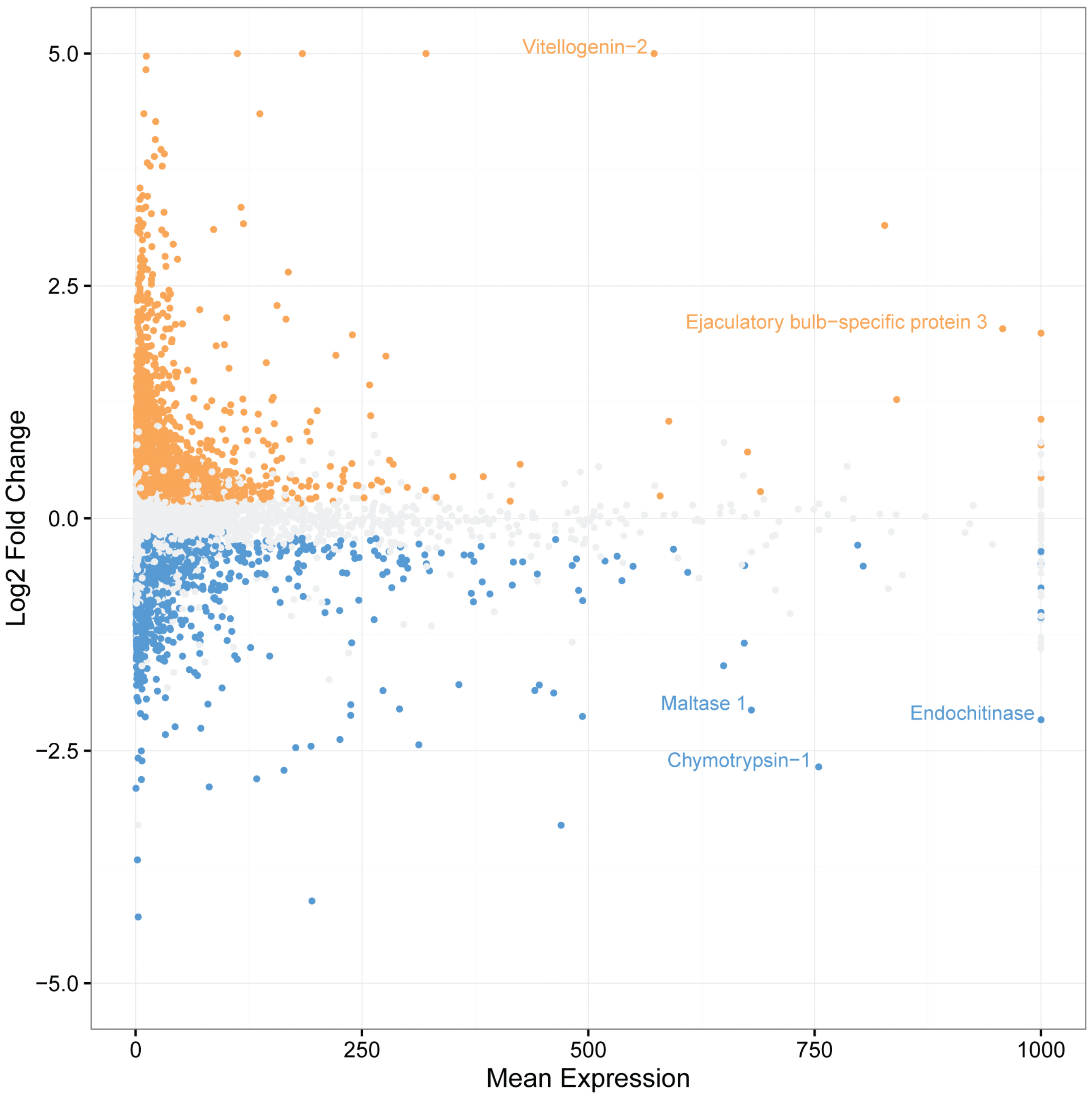

Vitellogenin−2
Ejaculatory bulb−specific protein 3
Maltase 1
Endochitinase
Chymotrypsin−1
5.0
2.5
0.0
−2.5
−5.0
Log2 Fold Change
0
250
500
750
1000
Mean Expression

Figure S3

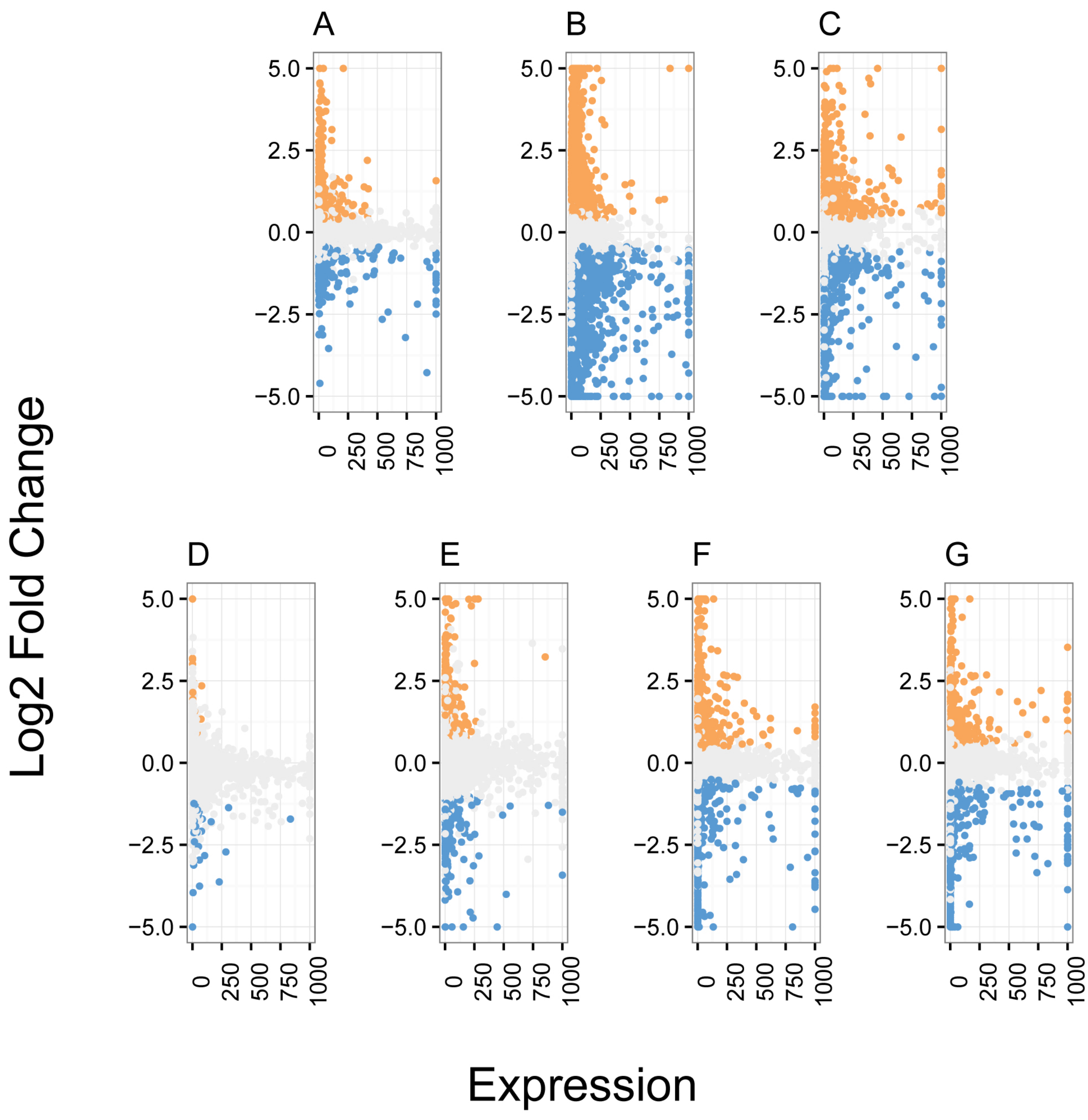

A
B
C
D
E
F
G
5.0
2.5
0.0
−2.5
−5.0
0
250
500
750
1000
Log2 Fold Change
Expression

Figure S4

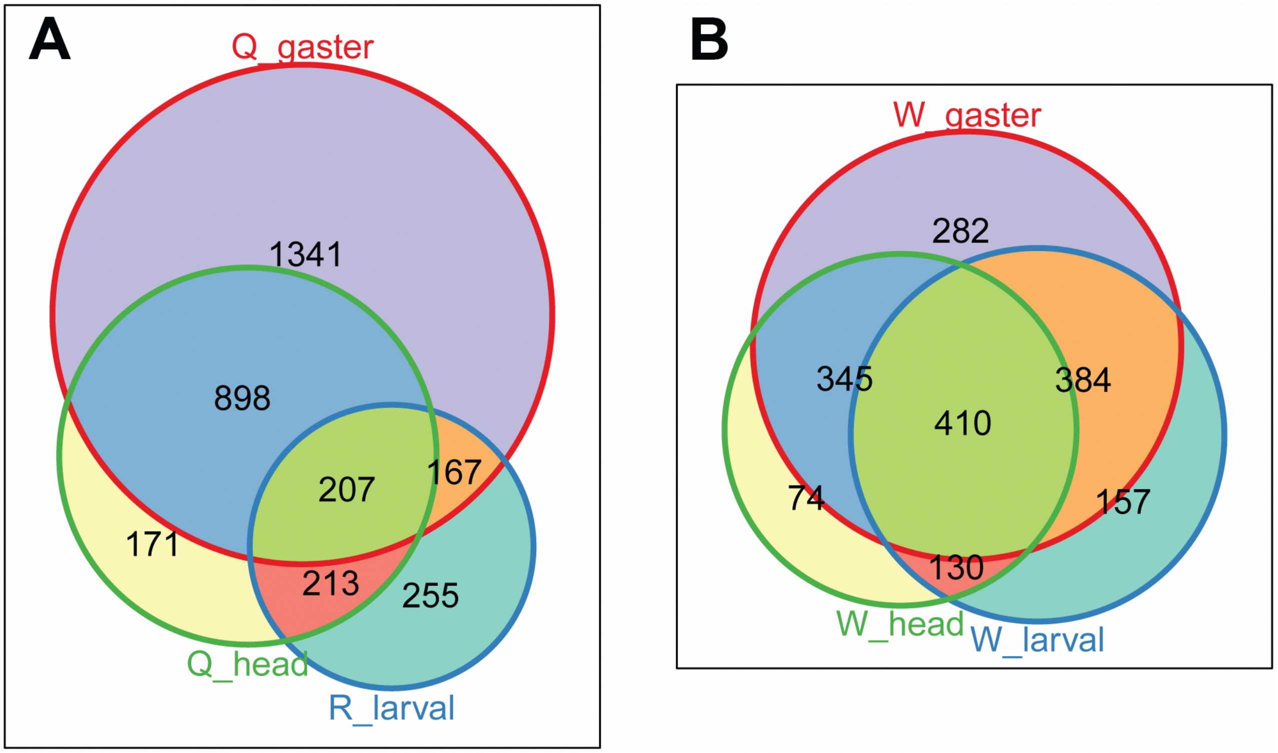
A
Q_gaster
1341
898
207
167
171
213
255
Q_head
R_larval
B
W_gaster
282
345
384
410
74
157
130
W_head
W_larval

Figure S5

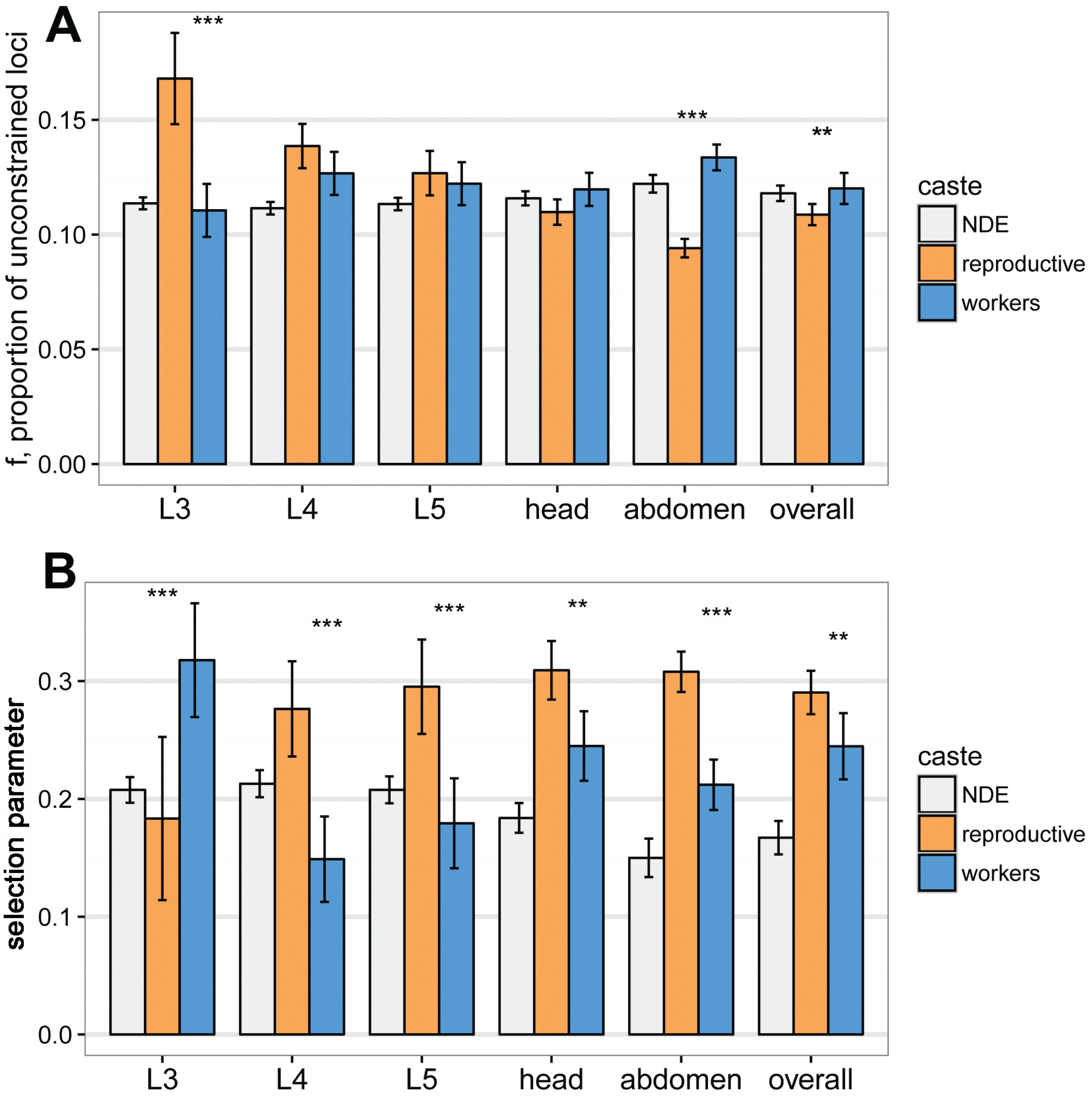
A
B
f, proportion of unconstrained loci
selection parameter
0.00
0.05
0.10
0.15
0.0
0.1
0.2
0.3
L3
L4
L5
head
abdomen
overall
***
**
caste
NDE
reproductive
workers

Figure S6

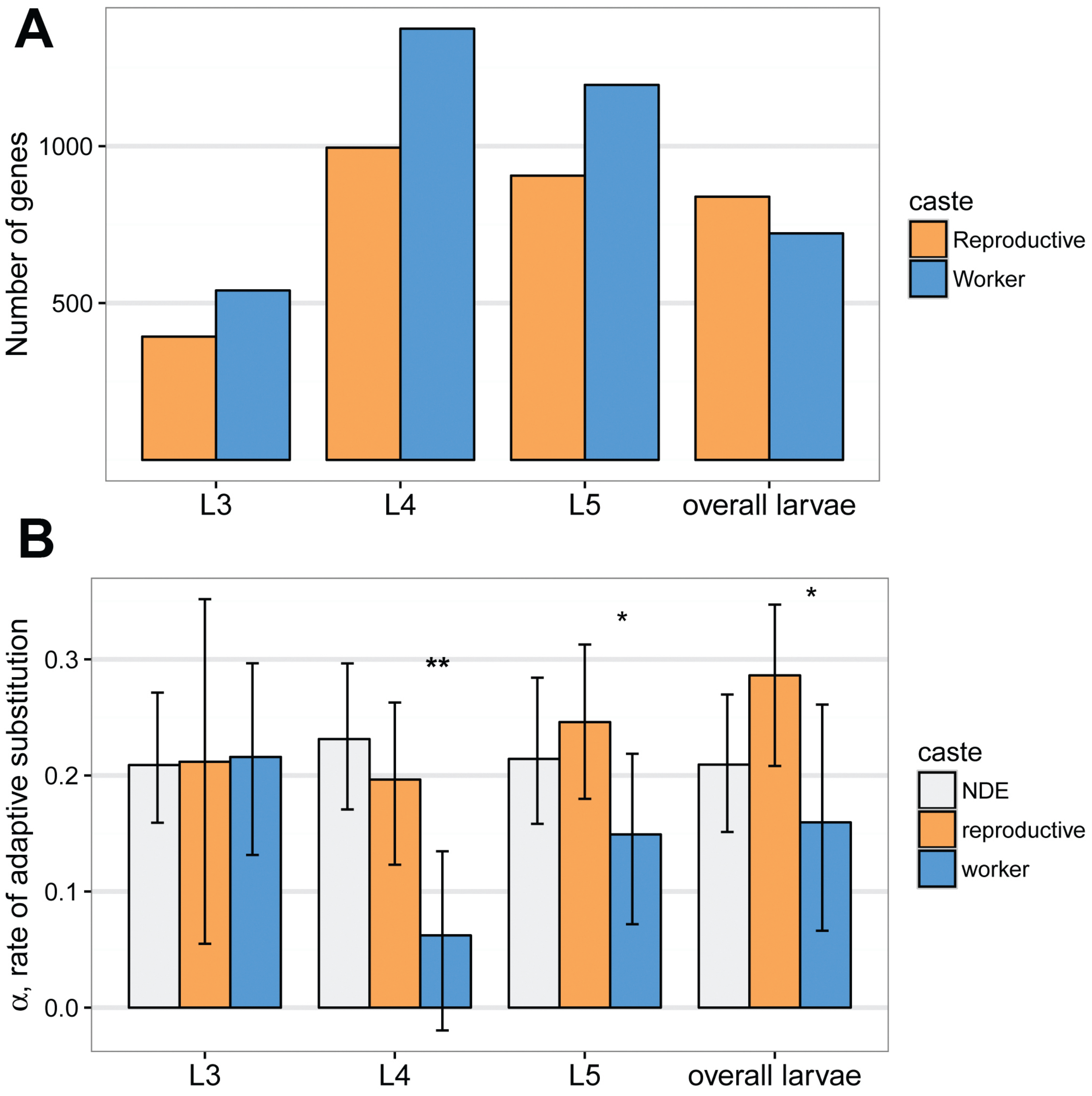
A
Number of genes
1000
500
L3
L4
L5
overall larvae
caste
Reproductive
Worker
B
α, rate of adaptive substitution
0.3
0.2
0.1
0.0
**
*
*
L3
L4
L5
overall larvae
caste
NDE
reproductive
worker

Figure S7

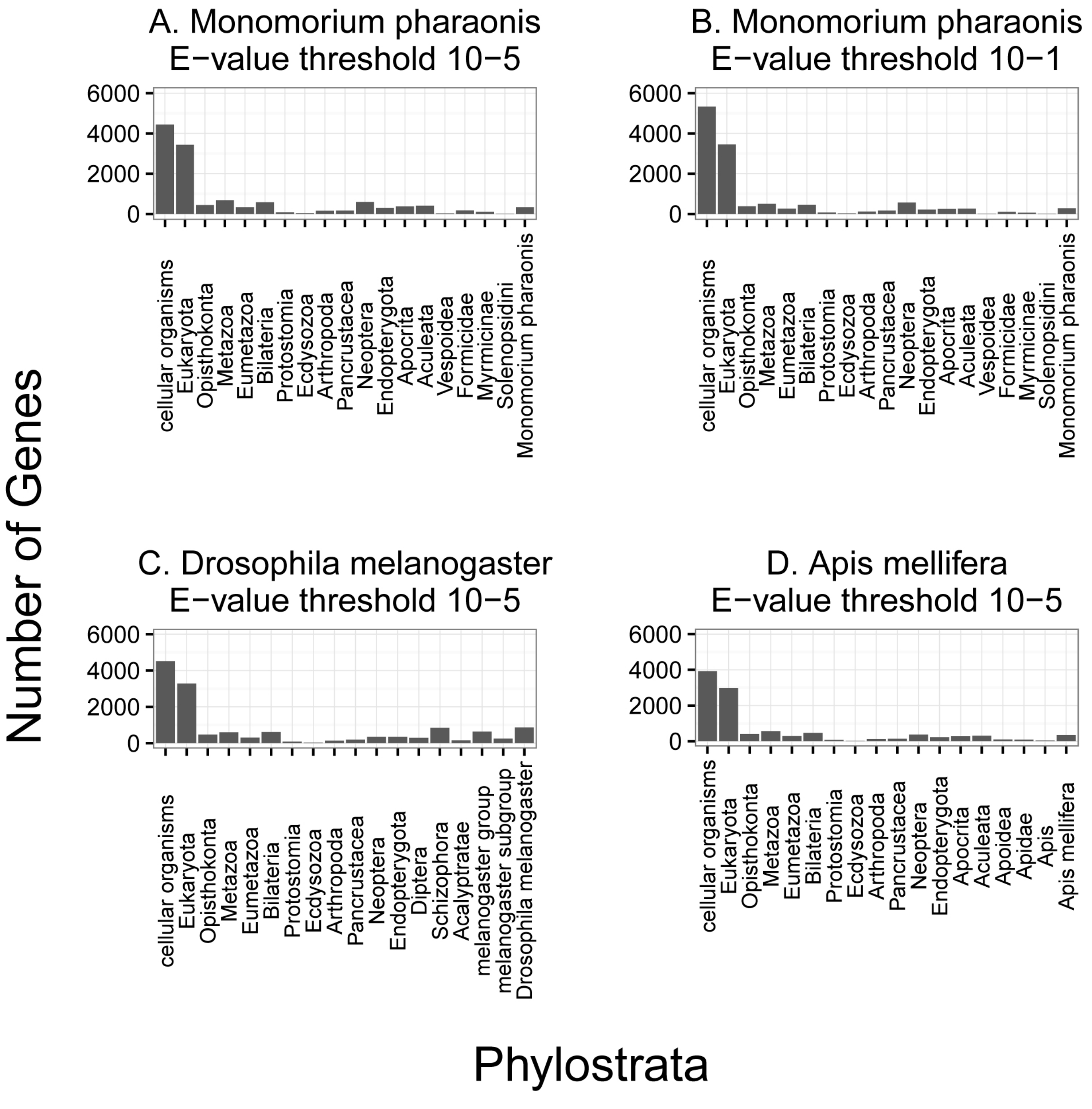
A. Monomorium pharaonis
E−value threshold 10−5
B. Monomorium pharaonis
E−value threshold 10−1
C. Drosophila melanogaster
E−value threshold 10−5
D. Apis mellifera
E−value threshold 10−5
Number of Genes
Phylostrata
6000
4000
2000
0
cellular organisms
Eukaryota
Opisthokonta
Metazoa
Eumetazoa
Bilateria
Protostomia
Ecdysozoa
Arthropoda
Pancrustacea
Neoptera
Endopterygota
Apocrita
Aculeata
Vespoidea
Formicidae
Myrmicinae
Solenopsidini
Monomorium pharaonis
Diptera
Schizophora
Acalyptratae
melanogaster group
melanogaster subgroup
Drosophila melanogaster
Apoidea
Apidae
Apis
Apis mellifera

Figure S8

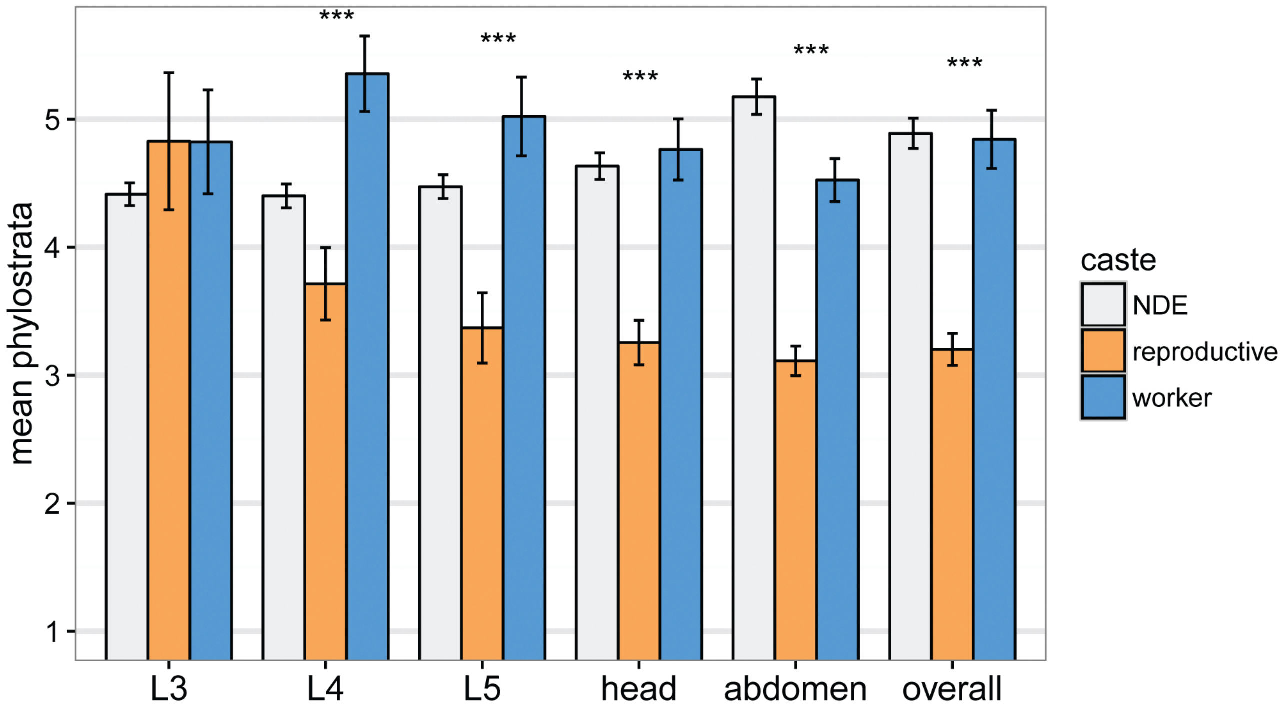

mean phylostrata
1
2
3
4
5
***
***
***
***
***
L3
L4
L5
head
abdomen
overall
caste
NDE
reproductive
worker

Figure S9

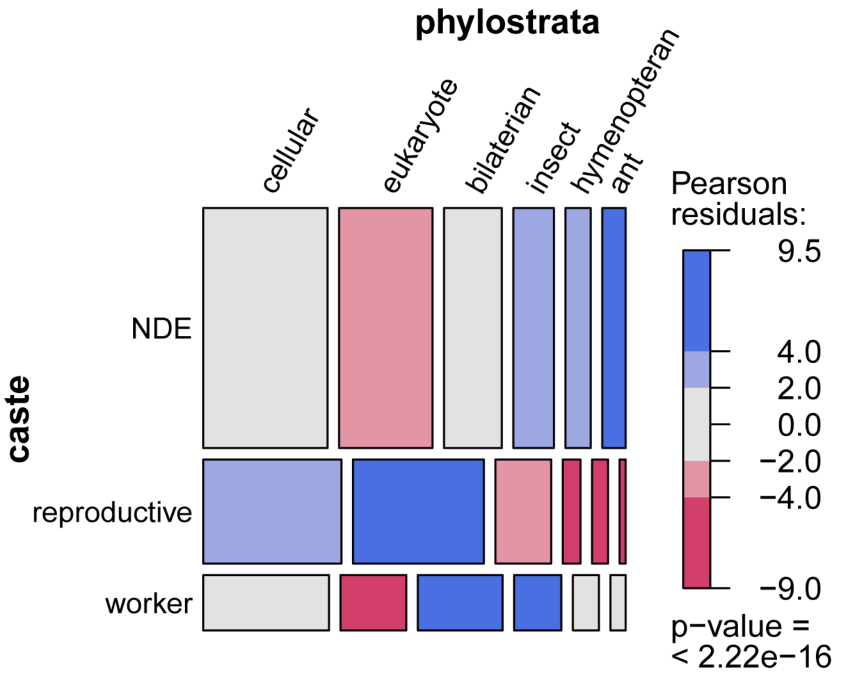
phylostrata
cellular
eukaryote
bilaterian
insect
hymenopteran
ant
caste
NDE
reproductive
worker
Pearson
residuals:
9.5
4.0
2.0
0.0
−2.0
−4.0
−9.0
p−value =
< 2.22e−16

Figure S10

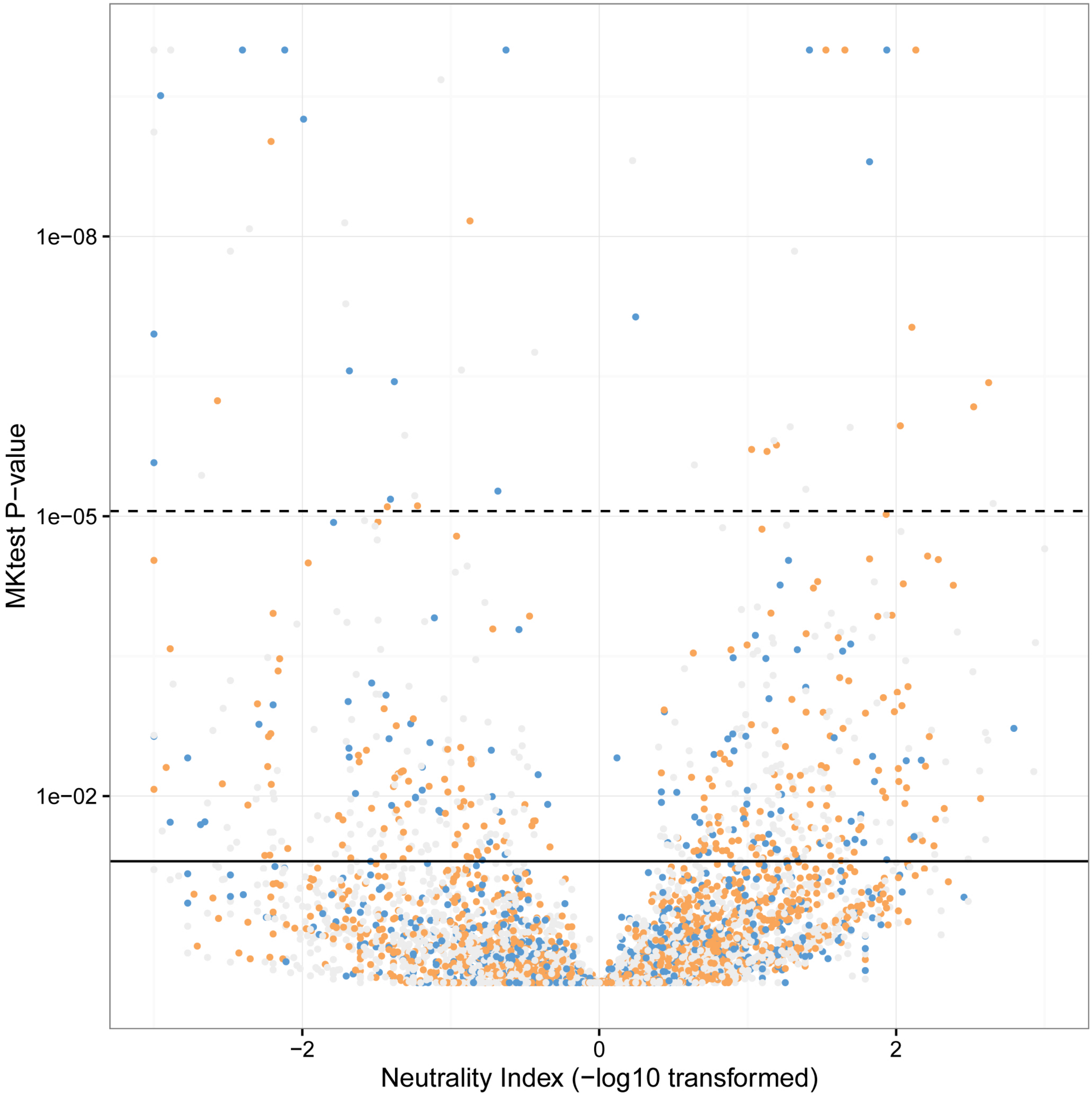

MKtest P−value
1e−08
1e−05
1e−02
−2
0
2
Neutrality Index (−log10 transformed)

Figure S11

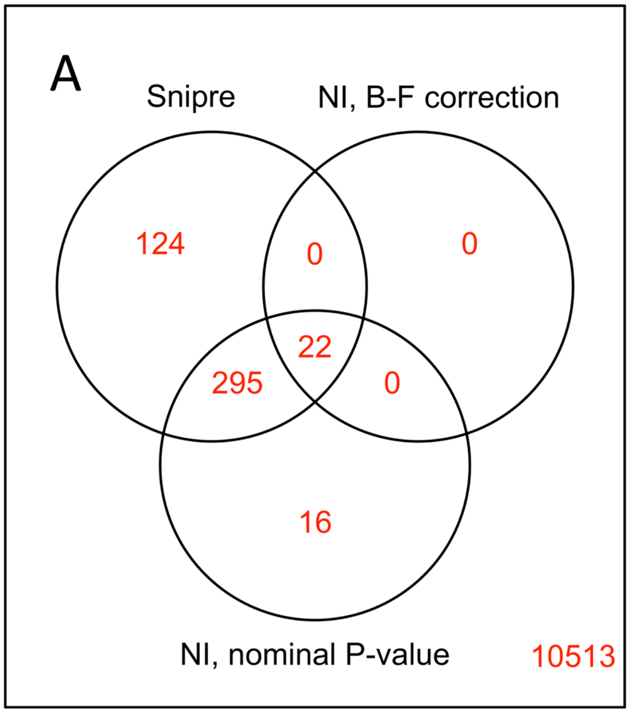
A
Snipre
NI, B-F correction
124
0
0
22
295
0
16
NI, nominal P-value
10513
B
Snipre
NI, B-F correction
30
0
0
30
112
0
105
NI, nominal P-value
10693

Table S1.

The number of biological replicates (out of three initial) for each sample type that were successfully analyzed (note that discrepancies between the study design shown in Figure S1 resulted from sample loss during processing). Please see Sampling Procedure and Fig. S1 for further details. Replicate colonies were assigned to a queen present or queen absent treatment ("Queen Presence" column) as well as to one of five time points ("L1" through "L5" columns). At each time point, larvae, nurses, and foragers were collected. Larvae from the L2-L5 time points were separated based on morphology into worker larvae and reproductive larvae, and we separately collected nurses that were observed feeding worker larvae (= "Worker Nurse") or reproductive larvae (= "Reproductive Nurse"). Whole larvae were processed, while the heads and gasters (=abdominal tissue) of nurses, foragers, and queens were processed separately. Reproductive larvae are not distinguishable at the L1 stage, hence the "N/A" for reproductive larvae and reproductive nurse samples at the L1 time point. Adult queen samples are in the "Other" column because they were collected separately, after the L1-L5 time points were collected, hence the "N/A" for L1-L5 for adult queen samples.

Sample	Queen Presence	L1	L2	L3	L4	L5	Other	Total
Worker Larva	Present	3	3	3	3	3	N/A	**15**
Worker Larva	Absent	3	3	3	3	3	N/A	**15**
Reproductive Larva	Absent	N/A	3	3	3	3	N/A	**12**
Forager Head	Present	1	3	3	1	1	N/A	**9**
Forager Gaster	Present	2	2	3	2	2	N/A	**11**
Forager Head	Absent	2	3	3	3	2	N/A	**13**
Forager Gaster	Absent	2	3	2	3	2	N/A	**12**
Worker Nurse Head	Present	3	3	3	2	2	N/A	**13**
Worker Nurse Gaster	Present	3	3	3	3	2	N/A	**14**
Worker Nurse Head	Absent	2	3	2	2	3	N/A	**12**
Worker Nurse Gaster	Absent	3	2	3	2	3	N/A	**13**
Reproductive Nurse Head	Absent	N/A	2	2	2	3	N/A	**9**
Reproductive Nurse Gaster	Absent	N/A	3	3	3	3	N/A	**12**
Adult Queen Head	Present	N/A	N/A	N/A	N/A	N/A	3	**3**
Adult Queen Gaster	Absent	N/A	N/A	N/A	N/A	N/A	2	**2**

Table S2.

Top 20 worker-upregulated genes, sorted by FDR. Differential expression calculated using glm-like model including caste and developmental stage as fixed effects. Negative values of Log2 fold change indicate higher expression in worker samples.

Gene	Log2 Fold Change	P-Value	FDR	Description (SwissProt)
**LOC105841041**	-1.83	2.62E-32	3.19E-30	-
**LOC105837944**	-1.39	2.13E-26	1.67E-24	-
**LOC105835450**	-0.83	4.82E-26	3.62E-24	-
**LOC105835451**	-1.03	5.34E-23	2.96E-21	Endothelin-converting enzyme 1
**LOC105834581**	-1.09	1.82E-22	9.57E-21	TPPP family protein CG45057
**LOC105832294**	-2.01	3.58E-22	1.82E-20	Phospholipase A1
**LOC105831727**	-1.29	3.82E-22	1.93E-20	Ras-related protein Rab-3
**LOC105836444**	-1.16	2.31E-21	1.11E-19	Neurotrimin
**LOC105831787**	-1.11	3.56E-20	1.51E-18	Zinc finger protein 362
**LOC105835899**	-1.35	5.20E-19	1.95E-17	CUGBP Elav-like family member 4
**LOC105835996**	-1.03	2.32E-18	8.36E-17	Glutamate-gated chloride channel
**LOC105831567**	-1.61	8.58E-18	2.89E-16	Transcription factor 21
**LOC105832152**	-1.73	1.72E-17	5.59E-16	Esterase E4
**LOC105837623**	-1.26	5.68E-17	1.71E-15	Lachesin
**LOC105832861**	-1.28	9.58E-17	2.77E-15	-
**LOC105837307**	-0.89	1.00E-16	2.89E-15	Ankyrin-2
**LOC105833207**	-1.39	2.45E-16	6.83E-15	Pikachurin
**LOC105829008**	-4.29	8.32E-16	2.21E-14	-
**LOC105830234**	-2.91	8.34E-16	2.21E-14	-
**LOC105830319**	-4.11	8.73E-16	2.30E-14	-

Table S3.

Top 20 reproductive-upregulated genes, sorted by FDR. Positive values of Log2 fold change indicate higher expression in reproductive samples.

Gene	Log2 Fold Change	P-Value	FDR	Description (SwissProt)
**LOC105837393**	5.96	1.63E-143	1.79E-139	-
**LOC105834006**	4.27	7.02E-106	3.85E-102	Gephyrin
**LOC105830579**	3.92	1.02E-95	3.72E-92	Cytoplasmic polyadenylation element-binding protein 1
**LOC105834706**	5.08	1.76E-89	4.83E-86	Maternal effect protein oskar
**LOC105835926**	7.37	4.12E-87	9.03E-84	Vitellogenin-2
**LOC105840630**	10.61	4.49E-79	8.21E-76	-
**LOC105840094**	4.83	3.34E-71	5.24E-68	RCC1 and BTB domain-containing protein 1
**LOC105831415**	2.52	3.38E-68	4.64E-65	-
**LOC105834586**	2.56	2.40E-67	2.92E-64	Putative bifunctional UDP-N-acetylglucosamine transferase and deubiquitinase ALG13
**LOC105828810**	2.77	5.75E-65	6.30E-62	Gephyrin
**LOC105829254**	2.81	2.21E-63	2.20E-60	Maternal protein exuperantia
**LOC105832526**	2.21	1.97E-62	1.80E-59	Protein aubergine
**LOC105830728**	3.29	5.56E-62	4.69E-59	S-phase kinase-associated protein 2
**LOC105833392**	4.08	7.20E-62	5.64E-59	Poly(A) RNA polymerase gld-2 homolog A
**LOC105828865**	3.28	7.20E-60	5.27E-57	Hyaluronan mediated motility receptor
**LOC105837552**	3.97	6.20E-58	4.25E-55	Ribonuclease H1
**LOC105829518**	2.18	1.06E-57	6.81E-55	-
**LOC105833500**	2.19	9.12E-57	5.56E-54	Serine/threonine-protein kinase Chk2
**LOC105838098**	2.29	1.10E-56	6.37E-54	-
**LOC105833023**	3.33	1.49E-56	8.19E-54	-

Table S4.

Summary of the raw phylostrata identified for genes in the *M. pharoanis* genome (Fig. S6), and 6 categories that phylostrata were grouped into "Condensed PS1". For some analyses that required ~100 genes in each caste-associated category, we also created a third grouping, "Condensed PS2" that combined the hymenopteran and ant categories.

PS	Condensed PS1	Condensed PS2
cellular organisms	cellular	cellular
Eukaryota	eukaryote	eukaryote
Opisthokonta	bilaterian	bilaterian
Metazoa	bilaterian	bilaterian
Eumetazoa	bilaterian	bilaterian
Bilateria	bilaterian	bilaterian
Protostomia	bilaterian	bilaterian
Ecdysozoa	insect	insect
Arthropoda	insect	insect
Pancrustacea	insect	insect
Neoptera	insect	insect
Endopterygota	insect	insect
Apocrita	hymenopteran	hymenopteran
Aculeata	hymenopteran	hymenopteran
Vespoidea	hymenopteran	hymenopteran
Formicidae	ant	hymenopteran
Myrmicinae	ant	hymenopteran
Solenopsidini	ant	hymenopteran
Monomorium pharaonis	ant	hymenopteran

Table S5.

Top 3 GO terms for workers and reproductives for each differential expression test, as calculated using the R package GOstats, sorted by p-value. L2 not included due to paucity of differentially expressed genes.

GOBPID	Pvalue	OddsRatio	ExpCount	Count	Size	Term	Sample	Caste
**GO:0006811**	1.95E-12	3.554038863	26.99482536	63	154	ion transport	MainEffect	Worker
**GO:0046034**	3.35E-10	20.61047619	3.681112549	17	21	ATP metabolic process	MainEffect	Worker
**GO:0007186**	6.95E-10	3.66109831	19.10672704	46	109	G-protein coupled receptor signaling pathway	MainEffect	Worker
**GO:0046483**	4.38E-29	2.926335878	172.740621	287	567	heterocycle metabolic process	MainEffect	Reproductive
**GO:0090304**	1.36E-28	3.148458605	141.0562743	246	463	nucleic acid metabolic process	MainEffect	Reproductive
**GO:1901360**	2.19E-28	2.8789485	173.9592497	287	571	organic cyclic compound metabolic process	MainEffect	Reproductive
**GO:0015711**	0.000725545	5.986786787	1.629366106	7	22	organic anion transport	LarvalMain	Worker
**GO:0015849**	0.000725545	5.986786787	1.629366106	7	22	organic acid transport	LarvalMain	Worker
**GO:0046942**	0.000725545	5.986786787	1.629366106	7	22	carboxylic acid transport	LarvalMain	Worker
**GO:1902578**	1.28E-05	2.016145186	36.39068564	61	388	single-organism localization	LarvalMain	Reproductive
**GO:0044699**	1.45E-05	1.69630845	140.6856404	175	1500	single-organism process	LarvalMain	Reproductive
**GO:0044765**	1.58E-05	2.009181745	35.82794308	60	382	single-organism transport	LarvalMain	Reproductive
**GO:0006040**	1.24E-06	7.450946644	2.302716688	12	40	amino sugar metabolic process	L3	Worker
**GO:0006030**	1.24E-06	7.450946644	2.302716688	12	40	chitin metabolic process	L3	Worker
**GO:1901071**	1.24E-06	7.450946644	2.302716688	12	40	glucosamine-containing compound metabolic process	L3	Worker
**GO:0006720**	1.96E-05	14.98484848	0.626778784	6	17	isoprenoid metabolic process	L3	Reproductive
**GO:0008299**	1.96E-05	14.98484848	0.626778784	6	17	isoprenoid biosynthetic process	L3	Reproductive
**GO:0044255**	0.000359857	4.730458221	2.285899094	9	62	cellular lipid metabolic process	L3	Reproductive
**GO:0007600**	7.99E-12	5.110028653	11.4851229	37	92	sensory perception	L4	Worker
**GO:0007606**	7.99E-12	5.110028653	11.4851229	37	92	sensory perception of chemical stimulus	L4	Worker
**GO:0003008**	7.99E-12	5.110028653	11.4851229	37	92	system process	L4	Worker
**GO:0044710**	1.73E-09	2.068259836	82.89844761	130	671	single-organism metabolic process	L4	Reproductive
**GO:0055114**	1.23E-08	2.372507113	40.52263907	75	328	oxidation-reduction process	L4	Reproductive

**GO:0005975**	9.96E-06	2.690731282	15.44307891	33	125	carbohydrate metabolic process	L4	Reproductive
**GO:0007608**	3.27E-09	6.209081836	6.119340233	23	53	sensory perception of smell	L5	Worker
**GO:0007600**	6.01E-07	3.551861702	10.62225097	28	92	sensory perception	L5	Worker
**GO:0007606**	6.01E-07	3.551861702	10.62225097	28	92	sensory perception of chemical stimulus	L5	Worker
**GO:0044710**	2.09E-08	1.99925	78.3412031	121	671	single-organism metabolic process	L5	Reproductive
**GO:0055114**	7.45E-08	2.305735369	38.29495472	70	328	oxidation-reduction process	L5	Reproductive
**GO:0008152**	0.000452937	1.509656659	233.6225744	262	2001	metabolic process	L5	Reproductive
**GO:0055114**	1.70E-26	3.543282815	119.3402329	209	328	oxidation-reduction process	Gaster	Worker
**GO:0044699**	7.12E-15	1.788020026	545.76326	649	1500	single-organism process	Gaster	Worker
**GO:0055085**	3.98E-14	2.877425945	81.50064683	135	224	transmembrane transport	Gaster	Worker
**GO:0044260**	7.56E-57	3.762404675	279.5653299	469	952	cellular macromolecule metabolic process	Gaster	Reproductive
**GO:0090304**	1.66E-56	5.213744618	135.9650712	286	463	nucleic acid metabolic process	Gaster	Reproductive
**GO:0006139**	1.18E-48	4.196444744	159.7516171	307	544	nucleobase-containing compound metabolic process	Gaster	Reproductive
**GO:0046034**	2.31E-10	21.1920078	3.599611902	17	21	ATP metabolic process	Head	Worker
**GO:0006163**	2.16E-09	7.100674262	7.027813713	24	41	purine nucleotide metabolic process	Head	Worker
**GO:0009161**	2.77E-09	14.11695906	3.942432083	17	23	ribonucleoside monophosphate metabolic process	Head	Worker
**GO:0006259**	9.13E-09	3.252254768	22.91332471	49	108	DNA metabolic process	Head	Reproductive
**GO:0044710**	4.50E-07	1.657178803	142.3596378	190	671	single-organism metabolic process	Head	Reproductive
**GO:0006950**	1.40E-06	3.418803419	14.42690815	32	68	response to stress	Head	Reproductive

Table S6.

Top 3 GO terms for each phylostrata category for each differential expression test, as calculated using the R package GOstats, sorted by p-value. L2 not included due to paucity of differentially expressed genes. Missing phylostrata categories returned no significant GO terms.

GOBPID	Pvalue	OddsRatio	ExpCount	Count	Size	Term	Sample	Caste	Phylostrata
**GO:0006811**	6.23E-12	4.075406342	16.13712807	46	154	ion transport	MainEffect	Worker	cellular
**GO:0044765**	2.17E-11	2.73200443	40.02846054	81	382	single-organism transport	MainEffect	Worker	cellular
**GO:0055085**	2.20E-11	3.324968041	23.47218629	57	224	transmembrane transport	MainEffect	Worker	cellular
**GO:0065007**	1.16E-06	3.287946429	14.76746442	33	593	biological regulation	MainEffect	Worker	eukaryote
**GO:0050789**	2.40E-06	3.19417122	14.46862872	32	581	regulation of biological process	MainEffect	Worker	eukaryote
**GO:0050794**	5.66E-06	3.074883684	14.26940492	31	573	regulation of cellular process	MainEffect	Worker	eukaryote
**GO:0007186**	1.46E-26	21.5191793	3.243208279	33	109	G-protein coupled receptor signaling pathway	MainEffect	Worker	bilaterian
**GO:0050794**	1.41E-23	9.089912281	17.04915912	60	573	regulation of cellular process	MainEffect	Worker	bilaterian
**GO:0050789**	3.06E-23	8.921545106	17.28719276	60	581	regulation of biological process	MainEffect	Worker	bilaterian
**GO:0006040**	6.78E-05	61.78378378	0.090556274	3	40	amino sugar metabolic process	MainEffect	Worker	insect
**GO:0006030**	6.78E-05	61.78378378	0.090556274	3	40	chitin metabolic process	MainEffect	Worker	insect
**GO:1901071**	6.78E-05	61.78378378	0.090556274	3	40	glucosamine-containing compound metabolic process	MainEffect	Worker	insect
**GO:0007600**	5.91E-39	152.978836	1.130659767	29	92	sensory perception	MainEffect	Worker	hymenopteran_ant
**GO:0007606**	5.91E-39	152.978836	1.130659767	29	92	sensory perception of chemical stimulus	MainEffect	Worker	hymenopteran_ant
**GO:0003008**	5.91E-39	152.978836	1.130659767	29	92	system process	MainEffect	Worker	hymenopteran_ant
**GO:0008152**	7.43E-29	3.538233934	349.4631307	456	2001	metabolic process	MainEffect	Reproductive	cellular
**GO:0044238**	3.11E-15	2.120088457	250.4398448	333	1434	primary metabolic process	MainEffect	Reproductive	cellular
**GO:0071704**	1.53E-13	2.023948498	262.839586	340	1505	organic substance metabolic process	MainEffect	Reproductive	cellular

**GO:0090304**	9.66E-15	3.036420958	45.0721216	95	463	nucleic acid metabolic process	MainEffect	Reproductive	eukaryote
**GO:0051649**	2.92E-12	5.605050139	9.345407503	34	96	establishment of localization in cell	MainEffect	Reproductive	eukaryote
**GO:0051641**	7.52E-12	5.190694127	10.12419146	35	104	cellular localization	MainEffect	Reproductive	eukaryote
**GO:0031323**	3.77E-17	9.529559748	5.68305304	32	191	regulation of cellular metabolic process	MainEffect	Reproductive	bilaterian
**GO:0019222**	1.56E-16	8.99047619	5.95084088	32	200	regulation of metabolic process	MainEffect	Reproductive	bilaterian
**GO:0060255**	2.58E-16	9.141108114	5.623544631	31	189	regulation of macromolecule metabolic process	MainEffect	Reproductive	bilaterian
**GO:0007600**	5.64E-10	123.4470588	0.26778784	7	92	sensory perception	MainEffect	Reproductive	hymenopteran_ant
**GO:0007606**	5.64E-10	123.4470588	0.26778784	7	92	sensory perception of chemical stimulus	MainEffect	Reproductive	hymenopteran_ant
**GO:0003008**	5.64E-10	123.4470588	0.26778784	7	92	system process	MainEffect	Reproductive	hymenopteran_ant
**GO:0006811**	1.39E-05	3.35645933	7.620310479	21	154	ion transport	LarvalMain	Worker	cellular
**GO:0006508**	0.000148191	2.454297821	13.01390686	27	263	proteolysis	LarvalMain	Worker	cellular
**GO:0006820**	0.000277076	5.735960591	1.781371281	8	36	anion transport	LarvalMain	Worker	cellular
**GO:0065007**	0.000350266	4.004145078	5.561772316	14	593	biological regulation	LarvalMain	Worker	eukaryote
**GO:0007165**	0.000783482	4.257379353	3.254527814	10	347	signal transduction	LarvalMain	Worker	eukaryote
**GO:0044700**	0.000820369	4.229156963	3.273285899	10	349	single organism signaling	LarvalMain	Worker	eukaryote
**GO:0050794**	4.20E-12	10.33104396	7.227360931	27	573	regulation of cellular process	LarvalMain	Worker	bilaterian
**GO:0050789**	5.93E-12	10.14936823	7.328266494	27	581	regulation of biological process	LarvalMain	Worker	bilaterian
**GO:0065007**	9.88E-12	9.886484099	7.479624838	27	593	biological regulation	LarvalMain	Worker	bilaterian

**GO:0006040**	0.025709996	78.23076923	0.025873221	1	40	amino sugar metabolic process	LarvalMain	Worker	insect
**GO:0006030**	0.025709996	78.23076923	0.025873221	1	40	chitin metabolic process	LarvalMain	Worker	insect
**GO:1901071**	0.025709996	78.23076923	0.025873221	1	40	glucosamine-containing compound metabolic process	LarvalMain	Worker	insect
**GO:0055085**	1.46E-06	2.87593985	14.63389392	34	224	transmembrane transport	LarvalMain	Reproductive	cellular
**GO:0044710**	3.85E-06	2.068575064	43.83635188	71	671	single-organism metabolic process	LarvalMain	Reproductive	cellular
**GO:0055114**	9.66E-05	2.153374233	21.42820181	39	328	oxidation-reduction process	LarvalMain	Reproductive	cellular
**GO:0006281**	2.68E-05	9.741395349	0.921733506	7	57	DNA repair	LarvalMain	Reproductive	eukaryote
**GO:0006974**	3.38E-05	9.360465116	0.954075032	7	59	cellular response to DNA damage stimulus	LarvalMain	Reproductive	eukaryote
**GO:0033554**	3.78E-05	9.180781044	0.970245796	7	60	cellular response to stress	LarvalMain	Reproductive	eukaryote
**GO:0006869**	3.01E-11	111.3072917	0.150388098	7	15	lipid transport	LarvalMain	Reproductive	bilaterian
**GO:0010876**	3.01E-11	111.3072917	0.150388098	7	15	lipid localization	LarvalMain	Reproductive	bilaterian
**GO:0033036**	1.54E-05	11.00949367	0.862225097	7	86	macromolecule localization	LarvalMain	Reproductive	bilaterian
**GO:0007600**	2.40E-05	45.40909091	0.208279431	4	92	sensory perception	LarvalMain	Reproductive	hymenopteran_ant
**GO:0007606**	2.40E-05	45.40909091	0.208279431	4	92	sensory perception of chemical stimulus	LarvalMain	Reproductive	hymenopteran_ant
**GO:0003008**	2.40E-05	45.40909091	0.208279431	4	92	system process	LarvalMain	Reproductive	hymenopteran_ant
**GO:0044765**	6.83E-06	2.752626552	15.19598965	33	382	single-organism transport	L3	Worker	cellular
**GO:1902578**	9.64E-06	2.699906103	15.43467012	33	388	single-organism localization	L3	Worker	cellular
**GO:0055114**	1.86E-05	2.754927773	13.04786546	29	328	oxidation-reduction process	L3	Worker	cellular

**GO:0030328**	0.004204398	Inf	0.004204398	1	1	prenylcysteine catabolic process	L3	Worker	eukaryote
**GO:0000098**	0.004204398	Inf	0.004204398	1	1	sulfur amino acid catabolic process	L3	Worker	eukaryote
**GO:0030329**	0.004204398	Inf	0.004204398	1	1	prenylcysteine metabolic process	L3	Worker	eukaryote
**GO:0007186**	4.68E-08	14.24723425	1.092820181	10	109	G-protein coupled receptor signaling pathway	L3	Worker	bilaterian
**GO:0050794**	9.65E-07	6.251975052	5.744825356	18	573	regulation of cellular process	L3	Worker	bilaterian
**GO:0050789**	1.20E-06	6.143462222	5.825032342	18	581	regulation of biological process	L3	Worker	bilaterian
**GO:0006040**	5.78E-09	217.8571429	0.090556274	5	40	amino sugar metabolic process	L3	Worker	insect
**GO:0006030**	5.78E-09	217.8571429	0.090556274	5	40	chitin metabolic process	L3	Worker	insect
**GO:1901071**	5.78E-09	217.8571429	0.090556274	5	40	glucosamine-containing compound metabolic process	L3	Worker	insect
**GO:0050909**	0.012613195	Inf	0.012613195	1	39	sensory perception of taste	L3	Worker	hymenopteran_ant
**GO:0007600**	0.029754204	Inf	0.029754204	1	92	sensory perception	L3	Worker	hymenopteran_ant
**GO:0007606**	0.029754204	Inf	0.029754204	1	92	sensory perception of chemical stimulus	L3	Worker	hymenopteran_ant
**GO:0006720**	4.38E-05	17.1347032	0.428848642	5	17	isoprenoid metabolic process	L3	Reproductive	cellular
**GO:0008299**	4.38E-05	17.1347032	0.428848642	5	17	isoprenoid biosynthetic process	L3	Reproductive	cellular
**GO:0044255**	0.000128384	6.264550265	1.564036223	8	62	cellular lipid metabolic process	L3	Reproductive	cellular
**GO:0010970**	0.005174644	Inf	0.005174644	1	1	establishment of localization by movement along microtubule	L3	Reproductive	eukaryote
**GO:0098840**	0.005174644	Inf	0.005174644	1	1	protein transport along microtubule	L3	Reproductive	eukaryote
**GO:0042073**	0.005174644	Inf	0.005174644	1	1	intraciliary transport	L3	Reproductive	eukaryote

**GO:0050794**	9.40E-06	20.08244681	2.038486417	9	573	regulation of cellular process	L3	Reproductive	bilaterian
**GO:0050789**	1.06E-05	19.73863636	2.06694696	9	581	regulation of biological process	L3	Reproductive	bilaterian
**GO:0065007**	1.26E-05	19.24058219	2.109637775	9	593	biological regulation	L3	Reproductive	bilaterian
**GO:0007600**	2.40E-06	43.04597701	0.26778784	5	92	sensory perception	L3	Reproductive	hymenopteran_ant
**GO:0007606**	2.40E-06	43.04597701	0.26778784	5	92	sensory perception of chemical stimulus	L3	Reproductive	hymenopteran_ant
**GO:0003008**	2.40E-06	43.04597701	0.26778784	5	92	system process	L3	Reproductive	hymenopteran_ant
**GO:0006508**	7.18E-09	3.162303099	18.11739974	44	263	proteolysis	L4	Worker	cellular
**GO:0006811**	3.41E-06	3.145542291	10.60866753	27	154	ion transport	L4	Worker	cellular
**GO:0055114**	3.00E-05	2.226843332	22.59508409	42	328	oxidation-reduction process	L4	Worker	cellular
**GO:0065007**	9.05E-11	5.535394265	12.08247089	35	593	biological regulation	L4	Worker	eukaryote
**GO:0050789**	1.43E-09	4.980109489	11.83796895	33	581	regulation of biological process	L4	Worker	eukaryote
**GO:0050794**	4.85E-09	4.747242263	11.67496766	32	573	regulation of cellular process	L4	Worker	eukaryote
**GO:0050794**	4.03E-19	11.27285115	11.30433376	43	573	regulation of cellular process	L4	Worker	bilaterian
**GO:0050789**	7.08E-19	11.0697026	11.46216041	43	581	regulation of biological process	L4	Worker	bilaterian
**GO:0065007**	1.62E-18	10.77606061	11.69890039	43	593	biological regulation	L4	Worker	bilaterian
**GO:0007600**	1.37E-51	274.8673469	1.279430789	36	92	sensory perception	L4	Worker	hymenopteran_ant
**GO:0007606**	1.37E-51	274.8673469	1.279430789	36	92	sensory perception of chemical stimulus	L4	Worker	hymenopteran_ant
**GO:0003008**	1.37E-51	274.8673469	1.279430789	36	92	system process	L4	Worker	hymenopteran_ant
**GO:0044710**	2.90E-13	2.638315484	63.1503881	115	671	single-organism metabolic process	L4	Reproductive	cellular
**GO:0055114**	2.52E-12	3.121996562	30.86934023	70	328	oxidation-reduction process	L4	Reproductive	cellular
**GO:0008152**	3.63E-09	2.293970547	188.3217982	232	2001	metabolic process	L4	Reproductive	cellular

**GO:0006810**	0.002909657	2.793736501	6.479301423	14	477	transport	L4	Reproductive	eukaryote
**GO:0051234**	0.003089857	2.772532189	6.520051746	14	480	establishment of localization	L4	Reproductive	eukaryote
**GO:0051179**	0.003687396	2.710526316	6.642302717	14	489	localization	L4	Reproductive	eukaryote
**GO:0006869**	1.81E-12	112.2949309	0.18919793	8	15	lipid transport	L4	Reproductive	bilaterian
**GO:0010876**	1.81E-12	112.2949309	0.18919793	8	15	lipid localization	L4	Reproductive	bilaterian
**GO:0033036**	7.86E-06	9.842845327	1.084734799	8	86	macromolecule localization	L4	Reproductive	bilaterian
**GO:0006040**	1.19E-07	339	0.064683053	4	40	amino sugar metabolic process	L4	Reproductive	insect
**GO:0006030**	1.19E-07	339	0.064683053	4	40	chitin metabolic process	L4	Reproductive	insect
**GO:1901071**	1.19E-07	339	0.064683053	4	40	glucosamine-containing compound metabolic process	L4	Reproductive	insect
**GO:0007600**	9.98E-05	101.0898876	0.119016818	3	92	sensory perception	L4	Reproductive	hymenopteran_ant
**GO:0007606**	9.98E-05	101.0898876	0.119016818	3	92	sensory perception of chemical stimulus	L4	Reproductive	hymenopteran_ant
**GO:0003008**	9.98E-05	101.0898876	0.119016818	3	92	system process	L4	Reproductive	hymenopteran_ant
**GO:0055114**	4.19E-08	2.723857659	23.97412678	51	328	oxidation-reduction process	L5	Worker	cellular
**GO:0006508**	7.65E-06	2.459845302	19.22315653	39	263	proteolysis	L5	Worker	cellular
**GO:0006811**	8.94E-05	2.638937097	11.25614489	25	154	ion transport	L5	Worker	cellular
**GO:0065007**	2.52E-10	6.7114595	9.205692109	29	593	biological regulation	L5	Worker	eukaryote
**GO:0050789**	9.91E-10	6.306329114	9.019404916	28	581	regulation of biological process	L5	Worker	eukaryote
**GO:0050794**	4.39E-09	5.882260597	8.895213454	27	573	regulation of cellular process	L5	Worker	eukaryote
**GO:0050794**	1.83E-13	10.65023374	7.968628719	30	573	regulation of cellular process	L5	Worker	bilaterian
**GO:0050789**	2.70E-13	10.46209689	8.079883571	30	581	regulation of biological process	L5	Worker	bilaterian
**GO:0065007**	4.77E-13	10.18991666	8.246765847	30	593	biological regulation	L5	Worker	bilaterian

**GO:0006040**	0.025709996	78.23076923	0.025873221	1	40	amino sugar metabolic process	L5	Worker	insect
**GO:0006030**	0.025709996	78.23076923	0.025873221	1	40	chitin metabolic process	L5	Worker	insect
**GO:1901071**	0.025709996	78.23076923	0.025873221	1	40	glucosamine-containing compound metabolic process	L5	Worker	insect
**GO:0007600**	4.56E-37	177.6065934	1.01164295	27	92	sensory perception	L5	Worker	hymenopteran_ant
**GO:0007606**	4.56E-37	177.6065934	1.01164295	27	92	sensory perception of chemical stimulus	L5	Worker	hymenopteran_ant
**GO:0003008**	4.56E-37	177.6065934	1.01164295	27	92	system process	L5	Worker	hymenopteran_ant
**GO:0044710**	2.40E-13	2.754534915	57.0740621	107	671	single-organism metabolic process	L5	Reproductive	cellular
**GO:0055114**	4.46E-13	3.363359137	27.89909444	67	328	oxidation-reduction process	L5	Reproductive	cellular
**GO:0008152**	1.75E-08	2.296387598	170.2014877	210	2001	metabolic process	L5	Reproductive	cellular
**GO:0051649**	0.000343296	5.356363636	1.800776197	8	96	establishment of localization in cell	L5	Reproductive	eukaryote
**GO:0022406**	0.000485846	27.52727273	0.168822768	3	9	membrane docking	L5	Reproductive	eukaryote
**GO:0051641**	0.000593227	4.896666667	1.95084088	8	104	cellular localization	L5	Reproductive	eukaryote
**GO:0006869**	1.05E-05	41.07744108	0.150388098	4	15	lipid transport	L5	Reproductive	bilaterian
**GO:0010876**	1.05E-05	41.07744108	0.150388098	4	15	lipid localization	L5	Reproductive	bilaterian
**GO:0050794**	0.000569083	3.685993897	5.744825356	14	573	regulation of cellular process	L5	Reproductive	bilaterian
**GO:0006040**	2.01E-06	Inf	0.038809832	3	40	amino sugar metabolic process	L5	Reproductive	insect

**GO:0006030**	2.01E-06	Inf	0.038809832	3	40	chitin metabolic process	L5	Reproductive	insect
**GO:1901071**	2.01E-06	Inf	0.038809832	3	40	glucosamine-containing compound metabolic process	L5	Reproductive	insect
**GO:0007600**	0.000244237	50.52808989	0.148771022	3	92	sensory perception	L5	Reproductive	hymenopteran_ant
**GO:0007606**	0.000244237	50.52808989	0.148771022	3	92	sensory perception of chemical stimulus	L5	Reproductive	hymenopteran_ant
**GO:0003008**	0.000244237	50.52808989	0.148771022	3	92	system process	L5	Reproductive	hymenopteran_ant
**GO:0055114**	5.29E-45	5.51156392	81.57567917	194	328	oxidation-reduction process	Gaster	Worker	cellular
**GO:0044710**	3.59E-28	2.829303501	166.8819534	280	671	single-organism metabolic process	Gaster	Worker	cellular
**GO:0055085**	7.96E-18	3.432707097	55.71021992	113	224	transmembrane transport	Gaster	Worker	cellular
**GO:0015991**	5.34E-07	44.71212121	0.401681759	6	9	ATP hydrolysis coupled proton transport	Gaster	Worker	eukaryote
**GO:0015988**	5.34E-07	44.71212121	0.401681759	6	9	energy coupled proton transmembrane transport, against electrochemical gradient	Gaster	Worker	eukaryote
**GO:0090662**	5.34E-07	44.71212121	0.401681759	6	9	ATP hydrolysis coupled transmembrane transport	Gaster	Worker	eukaryote
**GO:0007186**	4.14E-34	20.91978093	5.005821475	45	109	G-protein coupled receptor signaling pathway	Gaster	Worker	bilaterian
**GO:0050794**	3.55E-30	7.766867116	26.31500647	86	573	regulation of cellular process	Gaster	Worker	bilaterian
**GO:0065007**	7.35E-30	7.640244341	27.23350582	87	593	biological regulation	Gaster	Worker	bilaterian
**GO:0006040**	3.91E-05	82.40540541	0.077619664	3	40	amino sugar metabolic process	Gaster	Worker	insect
**GO:0006030**	3.91E-05	82.40540541	0.077619664	3	40	chitin metabolic process	Gaster	Worker	insect
**GO:1901071**	3.91E-05	82.40540541	0.077619664	3	40	glucosamine-containing compound metabolic process	Gaster	Worker	insect
**GO:0007600**	2.83E-72	265.3066202	1.934023286	51	92	sensory perception	Gaster	Worker	hymenopteran_ant
**GO:0007606**	2.83E-72	265.3066202	1.934023286	51	92	sensory perception of chemical stimulus	Gaster	Worker	hymenopteran_ant

**GO:0003008**	2.83E-72	265.3066202	1.934023286	51	92	system process	Gaster	Worker	hymenopteran_ant
**GO:0044237**	3.95E-32	3.340921765	174.6005821	290	1161	cellular metabolic process	Gaster	Reproductive	cellular
**GO:0044260**	6.29E-32	3.362369338	143.1694696	255	952	cellular macromolecule metabolic process	Gaster	Reproductive	cellular
**GO:0044238**	2.96E-26	2.997337956	215.656533	320	1434	primary metabolic process	Gaster	Reproductive	cellular
**GO:0090304**	2.39E-24	3.851938105	52.70892626	124	463	nucleic acid metabolic process	Gaster	Reproductive	eukaryote
**GO:0016070**	2.38E-19	3.639424649	41.55239327	99	365	RNA metabolic process	Gaster	Reproductive	eukaryote
**GO:0006139**	4.54E-18	3.050319094	61.9301423	125	544	nucleobase-containing compound metabolic process	Gaster	Reproductive	eukaryote
**GO:0050794**	4.03E-15	6.162917195	15.3813066	47	573	regulation of cellular process	Gaster	Reproductive	bilaterian
**GO:0050789**	7.11E-15	6.051029963	15.59605433	47	581	regulation of biological process	Gaster	Reproductive	bilaterian
**GO:0065007**	1.63E-14	5.889346764	15.91817594	47	593	biological regulation	Gaster	Reproductive	bilaterian
**GO:0007591**	0.000323415	Inf	0.000323415	1	1	molting cycle, chitin-based cuticle	Gaster	Reproductive	insect
**GO:0018990**	0.000323415	Inf	0.000323415	1	1	ecdysis, chitin-based cuticle	Gaster	Reproductive	insect
**GO:0022404**	0.000323415	Inf	0.000323415	1	1	molting cycle process	Gaster	Reproductive	insect
**GO:0007608**	9.18E-05	60.72	0.102846054	3	53	sensory perception of smell	Gaster	Reproductive	hymenopteran_ant
**GO:0007600**	0.000477944	33.6741573	0.178525226	3	92	sensory perception	Gaster	Reproductive	hymenopteran_ant
**GO:0007606**	0.000477944	33.6741573	0.178525226	3	92	sensory perception of chemical stimulus	Gaster	Reproductive	hymenopteran_ant
**GO:0044765**	1.70E-11	2.784824172	38.42238034	79	382	single-organism transport	Head	Worker	cellular

**GO:1902578**	3.97E-11	2.724137931	39.02587322	79	388	single-organism localization	Head	Worker	cellular
**GO:0006836**	3.03E-10	37.1638796	1.508732212	12	15	neurotransmitter transport	Head	Worker	cellular
**GO:0050789**	7.05E-08	3.619816514	15.2202458	36	581	regulation of biological process	Head	Worker	eukaryote
**GO:0065007**	1.22E-07	3.52459605	15.53460543	36	593	biological regulation	Head	Worker	eukaryote
**GO:0050794**	6.18E-07	3.317727865	15.0106727	34	573	regulation of cellular process	Head	Worker	eukaryote
**GO:0007186**	1.59E-27	25.41125541	2.820181113	32	109	G-protein coupled receptor signaling pathway	Head	Worker	bilaterian
**GO:0050794**	2.26E-22	9.976433971	14.82535576	54	573	regulation of cellular process	Head	Worker	bilaterian
**GO:0050789**	4.58E-22	9.793460809	15.03234153	54	581	regulation of biological process	Head	Worker	bilaterian
**GO:0006040**	2.59E-10	179.3529412	0.116429495	6	40	amino sugar metabolic process	Head	Worker	insect
**GO:0006030**	2.59E-10	179.3529412	0.116429495	6	40	chitin metabolic process	Head	Worker	insect
**GO:1901071**	2.59E-10	179.3529412	0.116429495	6	40	glucosamine-containing compound metabolic process	Head	Worker	insect
**GO:0007600**	3.24E-49	229.6491228	1.279430789	35	92	sensory perception	Head	Worker	hymenopteran_ant
**GO:0007606**	3.24E-49	229.6491228	1.279430789	35	92	sensory perception of chemical stimulus	Head	Worker	hymenopteran_ant
**GO:0003008**	3.24E-49	229.6491228	1.279430789	35	92	system process	Head	Worker	hymenopteran_ant
**GO:0008152**	6.79E-27	4.180646933	262.097348	352	2001	metabolic process	Head	Reproductive	cellular
**GO:0044710**	3.33E-15	2.509660566	87.88971539	152	671	single-organism metabolic process	Head	Reproductive	cellular
**GO:0055114**	1.44E-08	2.325211009	42.96248383	78	328	oxidation-reduction process	Head	Reproductive	cellular
**GO:0007017**	1.05E-07	8.821647059	2.249029754	13	38	microtubule-based process	Head	Reproductive	eukaryote
**GO:0006259**	7.08E-07	4.204767986	6.391979301	21	108	DNA metabolic process	Head	Reproductive	eukaryote
**GO:0033554**	1.09E-06	5.682539683	3.551099612	15	60	cellular response to stress	Head	Reproductive	eukaryote
**GO:0065007**	5.29E-08	4.252502224	12.08247089	31	593	biological regulation	Head	Reproductive	bilaterian

**GO:0050789**	1.38E-07	4.088434252	11.83796895	30	581	regulation of biological process	Head	Reproductive	bilaterian
**GO:0050794**	4.08E-07	3.896247837	11.67496766	29	573	regulation of cellular process	Head	Reproductive	bilaterian
**GO:0048598**	0.001293661	Inf	0.001293661	1	1	embryonic morphogenesis	Head	Reproductive	hymenopteran_ant
**GO:0035434**	0.003877217	514.3333333	0.003880983	1	3	copper ion transmembrane transport	Head	Reproductive	hymenopteran_ant
**GO:0009790**	0.003877217	514.3333333	0.003880983	1	3	embryo development	Head	Reproductive	hymenopteran_ant

Table S7.

Model selection parameters from MKtest2.0 (*13, 14*) for estimating *α*, the proportion of amino acid substitution driven by positive selection. The first three columns show the number of parameters for *α* and *f*, as well as the total number of model parameters, *K*. We mainly considered models with per-class estimates (i.e. three separate estimates for worker-associated, reproductive-associated, and NDE genes) for both *α* and *f*, or with per-locus estimates for *f*. Of these two main models in bold that we considered, the model including per-locus estimates of *f* fit the data much better. We focus on results from this model, although the per-class *α* and *f* model produced very similar results, showing the same pattern and overlapping *α* estimates. We also show results from models where *α* and/or *f* is fixed or had a single, genome-wide estimate. LnL maximized log likelihood; AIC, Akaike information criterion; AICc, second-order AIC; BIC, Bayesian information criterion (Welch 2006; Obbard et al. 2009).

Model description	*α*	*f*	*K*	*LnL*	AIC	AICc	BIC
	0	0	2	-1002096.22231	2004196.44462	2004196.44495	2004213.43161
	3	0	5	-678989.00409	1357988.00818	1357988.00984	1358030.4765
	1	0	3	-615656.376167	1231318.75233	1231318.753	1231344.23282
	0	1	3	-522404.908371	1044815.81674	1044815.81741	1044841.29722
	1	1	4	-521956.917522	1043921.83504	1043921.83615	1043955.80902
	1	3	6	-521585.629106	1043183.25821	1043183.26054	1043234.21918
	3	1	6	-521394.185871	1042800.37174	1042800.37407	1042851.33271
**Per-class α and f**	**3**	**3**	**8**	**-521349.0028**	**1042714.0056**	**1042714.00959**	**1042781.95355**
	0	9020	9022	-378721.983142	775487.966284	781505.300505	852116.268919
	1	9020	9023	-377650.16441	773346.32882	779365.219417	849983.124949
**Per-class α and per-locus f**	**3**	**9020**	**9025**	**-377432.859261**	**772917.718522**	**778937/722662**	**849569.501639**

Table S8.

Top 20 positively selected genes (sorted by p-value of McDonald-Kreitman test) for reproductive- and worker-associated genes. SnIPRE.class is the selection categories as calculated by SnIPRE. "NI.class" refers to selection categories, as calculated using a combination of the neutrality index and the P-value from the McDonald-Kreitman test. Genes with negative values of -log10(Neutrality Index) and p-values less than 0.05 are defined as under purifying selection, while such genes with positive -log10(Neutrality Index) values are assigned to the positive selection category. "NI.class B-F correction" uses the same method but the p-value cutoff from the McDonald-Kreitman test is adjusted for multiple comparisons using the Bonferroni procedure.

Gene	Snipre. class	NI.class	NI.class (B-F correction)	Gamma	Neutrality Index (-Log10 tranformed)	MK P-value	Description (SwissProt)	Description (UniProt)	Caste
**LOC105832526**	pos	pos	pos	5.94	2.13	8.70E-15	Protein aubergine	Piwi-like protein	Reproductive
**LOC105828570**	pos	pos	pos	3.24	1.53	3.76E-12	ABC transporter G family member 20	ABC transporter G family member 20 (Fragment)	Reproductive
**LOC105829311**	pos	pos	pos	5.53	2.52	6.69E-07	Probable ATP-dependent RNA helicase spindle-E	Putative ATP-dependent RNA helicase TDRD9	Reproductive
**LOC105836534**	pos	pos	pos	2.43	1.29	1.10E-06	Probable multidrug resistance-associated protein lethal(2)03659	Putative multidrug resistance-associated protein lethal(2)03659	Reproductive
**LOC105830831**	pos	pos	pos	2.22	1.18	1.54E-06	Transient receptor potential cation channel subfamily V member 5	Putative uncharacterized protein (Fragment)	Reproductive
**LOC105831051**	pos	pos	pos	2.05	1.13	2.02E-06	Venom serine protease Bi-VSP	Uncharacterized protein	Reproductive
**LOC105831878**	pos	pos	neut	4.05	2.21	2.67E-05	Adenylate cyclase type 8	Uncharacterized protein	Reproductive
**LOC105833310**	pos	pos	neut	3.54	1.82	2.86E-05	Structural maintenance of	Structural maintenance of	Reproductive

							chromosomes protein 5	chromosomes protein 5	
**LOC105835371**	pos	pos	neut	4.34	2.28	2.91E-05	Major facilitator superfamily domain-containing protein 6	Major facilitator superfamily domain-containing protein 6 (Fragment)	Reproductive
**LOC105828996**	pos	pos	neut	3.46	2.38	5.48E-05	-	-	Reproductive
**LOC105832368**	pos	pos	neut	1.97	1.16	0.000109 152	Biotin--protein ligase	Biotin--protein ligase (Fragment)	Reproductive
**LOC105840269**	pos	pos	neut	4.02	2.01	0.000771 347	-	Putative uncharacterized protein (Fragment)	Reproductive
**LOC105833118**	pos	pos	neut	1.74	1.12	0.003593 458	Serine/threonine-protein kinase SIK2	Uncharacterized protein	Reproductive
**LOC105837631**	pos	pos	neut	1.32	0.84	0.004018 889	Luciferin 4-monooxygenase	Luciferin 4-monooxygenase	Reproductive
**LOC105837563**	pos	pos	neut	1.46	0.88	0.004464 27	Probable multidrug resistance-associated protein lethal(2)03659	Multidrug resistance-associated protein 4	Reproductive
**LOC105830185**	pos	pos	neut	2.08	1.43	0.005106 672	-	Putative uncharacterized protein	Reproductive
**LOC105835393**	pos	pos	neut	2.33	1.88	0.005291 519	Serine protease snake	Serine protease snake	Reproductive
**LOC105830282**	pos	pos	neut	0.61	0.42	0.005630 685	Lymphoid-restricted membrane protein	Protein SAAL1 (Fragment)	Reproductive
**LOC105835118**	pos	pos	neut	1.78	1.35	0.005987 065	-	Putative uncharacterized protein (Fragment)	Reproductive

**LOC105837746**	pos	pos	neut	1.10	0.74	0.006518212	ATP-binding cassette sub-family A member 13	ATP-binding cassette sub-family A member 13	Reproductive
**LOC105833674**	pos	pos	pos	4.99	1.94	1.44E-14	Voltage-dependent calcium channel type A subunit alpha-1	Voltage dependent Ca2 channel Cav2 subunit	Worker
**LOC105835213**	pos	pos	pos	3.01	1.42	7.85E-12	Protein unc-79 homolog	Uncharacterized protein	Worker
**LOC105837842**	pos	pos	pos	4.23	1.82	1.58E-09	Sodium-independent sulfate anion transporter	Sodium-independent sulfate anion transporter	Worker
**LOC105832885**	pos	pos	neut	2.33	1.27	2.97E-05	Talin-1	Talin-1	Worker
**LOC105835926**	pos	pos	neut	2.66	1.61	0.000200458	Vitellogenin-2	Putative uncharacterized protein (Fragment)	Worker
**LOC105840699**	pos	pos	neut	2.03	1.17	0.000508296	Vitamin K-dependent gamma-carboxylase	Uncharacterized protein	Worker
**LOC105837307**	pos	pos	neut	0.66	0.44	0.00124757	Ankyrin-2	Putative uncharacterized protein (Fragment)	Worker
**LOC105838219**	pos	pos	neut	3.38	2.79	0.001877545	Glutamate receptor ionotropic kainate 2	Glutamate receptor ionotropic kainate 2	Worker
**LOC105836662**	pos	pos	neut	1.51	0.96	0.002224554	Protein phosphatase 1E	Protein phosphatase 1F	Worker
**LOC105831090**	pos	pos	neut	2.83	2.08	0.003082822	Dipeptidyl peptidase 3	Putative uncharacterized protein (Fragment)	Worker

**LOC105837460**	pos	pos	neut	1.32	0.78	0.005261444	-	Putative uncharacterized protein	Worker
**LOC105834708**	pos	pos	neut	1.73	1.16	0.005502118	Glucose dehydrogenase FAD quinone	Uncharacterized protein	Worker
**LOC105837257**	pos	pos	neut	1.54	0.99	0.012181701	Periaxin	Putative uncharacterized protein	Worker
**LOC105837961**	pos	pos	neut	1.46	0.97	0.014182719	Protein disconnected	Putative uncharacterized protein (Fragment)	Worker
**LOC105828589**	pos	pos	neut	1.74	1.22	0.014358752	Harmonin	Uncharacterized protein	Worker
**LOC105832072**	pos	pos	neut	1.56	1.16	0.015659886	-	Putative uncharacterized protein	Worker
**LOC105829245**	pos	pos	neut	2.43	1.76	0.015757911	Discoidin domain-containing receptor 2	Putative uncharacterized protein (Fragment)	Worker
**LOC105835954**	pos	pos	neut	1.03	0.64	0.016746237	SH3 and multiple ankyrin repeat domains protein 3	SH3 and multiple ankyrin repeat domains protein 3	Worker
**LOC105840696**	pos	pos	neut	1.97	1.21	0.017236477	Cytochrome P450 4C1	Cytochrome P450 4C1	Worker
**LOC105831887**	pos	pos	neut	2.15	1.76	0.01743138	Lysine-specific demethylase 6A	Putative uncharacterized protein (Fragment)	Worker

External Database S1.

Complete list of genes summarizing the per-locus results of differential expression analyses, population genomic analyses, and phylostratigraphy analyses. Columns show: annotation from SwissProt and UniProt; results from differential expression analysis by larval stage (L2-L5), adult head and gaster (abdominal) tissue, across all larval samples, and across all samples, with levels NDE = non differentially expressed genes, Reproductive = reproductive-upregulated, and Worker = worker-upregulated; counts of nonsynonymous and synonymous polymorphisms within *M. pharaonis* and fixed differences between *M. pharaonis* and *M. chinense*, and total numbers of nonsynonymous and synonymous sites; results from SnIPRE analysis including BSnIPRE.class, whether genes are categorized by SnIPRE as experiencing positive selection (“pos”), negative selection (“neg”), or neither (“neut”), BSnIPRE.gamma, a population-size calibrated selection coefficient estimate, and BSnIPRE.est, normalized BSnIPRE.gamma; “NI.class” refers to selection categories, as calculated using a combination of the neutrality index and the p-value from the McDonald-Kreitman test: Genes with negative values of -log10(Neutrality Index) and p-values less than 0.05 are defined as under purifying selection, while such genes with positive -log10(Neutrality Index) values are assigned to the positive selection category. “NI.class B-F correction” uses the same method but the p-value cutoff from the McDonald-Kreitman is adjusted for multiple comparisons using the Bonferroni procedure; Finally, the assigned raw (“Raw PS”) and condensed phylostrata (“PS1” and “PS2”; Table S4) from the phylostratigraphy analyses are shown.

External Database S2.

Complete GO enrichment analysis results for workers and reproductives for each differential expression test, as calculated using the R package GOstats, sorted by p-value. L2 not included due to paucity of differentially expressed genes.

External Database S3.

Complete GO enrichment analysis results for each phylostrata category for each differential expression test, as calculated using the R package GOstats, sorted by p-value. L2 not included due to paucity of differentially expressed genes. Missing phylostrata categories returned no significant GO terms.

External Database S4.

Raw counts per locus from RNA sequencing showing level of expression across all samples included in the study (Table S1).

External Database S5.

Raw FPKM per locus from RNA sequencing showing level of expression across all samples included in the study (Table S1).
